# Nanophotonic-enhanced photoacoustic imaging for brain tumor detection

**DOI:** 10.1186/s12951-025-03204-5

**Published:** 2025-03-05

**Authors:** Ali Rizwan, Badrinathan Sridharan, Jin Hyeong Park, Daehun Kim, Jean-Claude Vial, Kwangseuk Kyhm, Hae Gyun Lim

**Affiliations:** 1 Smart Gym-Based Translational Research Center for Active Senior’S Healthcare, Pukyong National University, Busan, 48513 Republic of Korea; 2 Department of Biomedical Engineering, Pukyong National University, Busan, 48513 Republic of Korea; 3 Indusrty 4.0 Convergence Bionics Engineering, Pukyong National University, Busan, 48513 Republic of Korea; 4 Université Grenoble Alpes, CNRS, LIPhy, 38000 Grenoble, France; 5 Department of Optics & Cogno-Mechatronics Engineering, Pusan National University, Busan, 46241 Republic of Korea

**Keywords:** Nanophotonics, Ultrasound, Photoacoustic brain imaging, Blood–brain barrier

## Abstract

Photoacoustic brain imaging (PABI) has emerged as a promising biomedical imaging modality, combining high contrast of optical imaging with deep tissue penetration of ultrasound imaging. This review explores the application of photoacoustic imaging in brain tumor imaging, highlighting the synergy between nanomaterials and state of the art optical techniques to achieve high-resolution imaging of deeper brain tissues. PABI leverages the photoacoustic effect, where absorbed light energy causes thermoelastic expansion, generating ultrasound waves that are detected and converted into images. This technique enables precise diagnosis, therapy monitoring, and enhanced clinical screening, specifically in the management of complex diseases such as breast cancer, lymphatic disorder, and neurological conditions. Despite integration of photoacoustic agents and ultrasound radiation, providing a comprehensive overview of current methodologies, major obstacles in brain tumor treatment, and future directions for improving diagnostic and therapeutic outcomes. The review underscores the significance of PABI as a robust research tool and medical method, with the potential to revolutionize brain disease diagnosis and treatment.

**Graphical Abstract:**

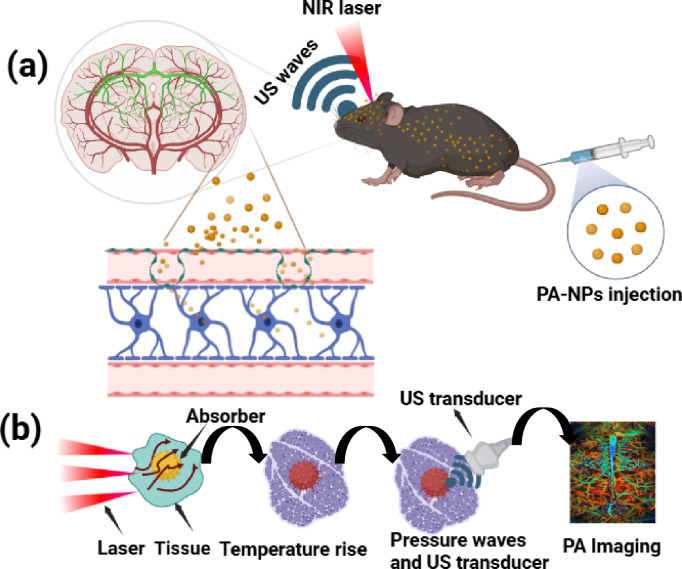

## Introduction

Nanophotonics involves the manipulation of light-matter interactions at the nanoscale, encompassing the investigation of new materials, optical interactions, manufacturing processes, and the development of inorganic/organic nanomaterials, and chemically synthesized materials as like quantum dots (QDs), sub-wavelength structures, plasmonic and photonic crystals, holey fibers, presents both scientific and technological challenges [1, 2]. In particular, the detection, prevention, and treatment of disease using photonics have become rapidly developing and ground breaking technologies [3]. Thanks to remotely accessible optical operations with extremely high-speed light modulation, light can be used to enhance diagnosis and drug production. This has led to significant advancements in specific diagnostic techniques, enabling improved tracking of patient therapy and clinical screenings [4, 5].

Photoacoustic imaging (PAI) combines the excellent contrast of traditional optical imaging with the exceptional spatiotemporal resolution of ultrasound (US) imaging, making it a remarkable biomedical imaging modality [6–8]. The phenomenon known as PAI effect was first discovered by Alexander Graham Bell in the year 1880. Absorbed light energy leads to quick thermoelastic expansion of biological structures, leading to creation of a US wave that can be identified by a US transducer and transformed into electric signals for processing into a PA image. PAI relies on the light-absorption coefficient of the tissue being imaged, without the need for invasive methods. Because the PA signals changes based on how light is absorbed in biological tissue, PA imaging is akin to optical imaging, offering enhanced imaging contrast and tissue sensitivity. Nonetheless, the result transmitter of the light-absorption data is not interpreted as an optical signal but as a US wave signal transformed into an image, with the advantages of minimal scattering and loss in biological tissue. As a result, PA imaging combines the contrast obtained in the optical imaging with deep tissue penetration of US [9]. Therefore, PA has the capability to image tissue at greater depths than other optical imaging techniques [10, 11].

**Fig. 1:**
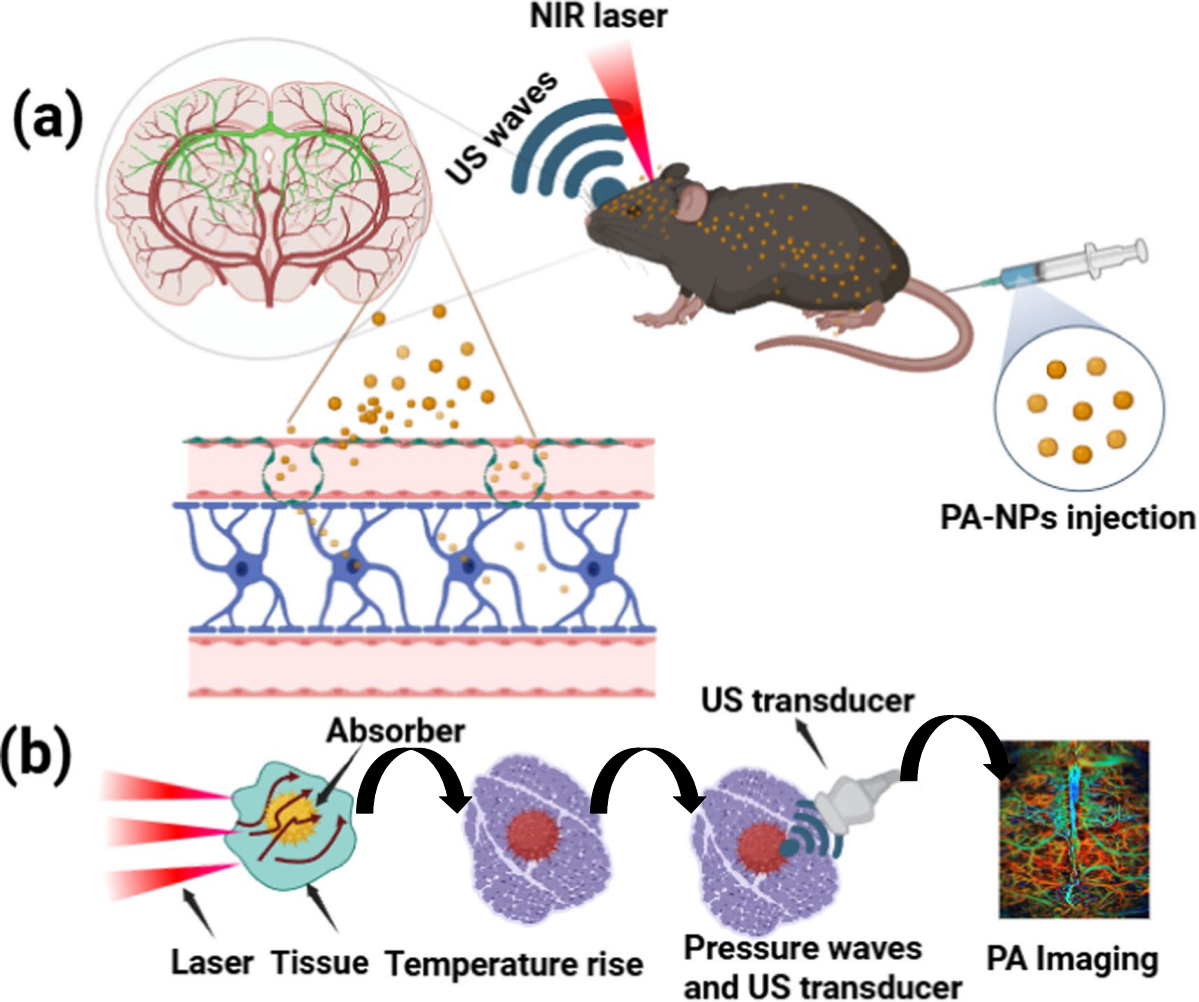
**a** Schematic illustration of the photoacoustic imaging of brain tumor and cross the PA agents into blood brain barrier (BBB); **b** basic principle of photoacoustic imaging. Created with BioRender.com

The synergy between light-matter interactions at nanoscale and PA imaging grasps tremendous capability in the field of biomedical applications. By leveraging nanomaterials and advanced optical techniques, PA imaging can achieve high-resolution images of deeper tissues, surpassing the limitations of traditional optical imaging technologies and has been illustrated in Fig. 1a. Consequently, it enables precise diagnosis and prognosis of many complex diseases, facilitates therapy monitoring, and enhances clinical screening, thereby driving significant advancements in healthcare. Despite the impressive advancements in PA technology, experts still find it challenging to clinically apply them in treating complex diseases. Photoacoustic (PA) imaging in a clinical set up predominantly aided in visualizing blood vessels and measuring blood oxygenation levels with support from endogenous contrast agents, such as hemoglobin and melanin, which exhibit strong near-infrared (NIR) absorption properties. These agents enable the visualization of angiogenesis in the cancer microenvironment, atherosclerotic plaques, and vascular tissue composition. However, PAI has certain limitations for diagnosis of diseases with complex pathophysiological mechanisms, due to lack of endogenous agents and this also complicates imaging certain systems, such as the lymphatic system or deep-seated organs. Invasiveness due to laser exposure in PA imaging is a notable factor and Laser intensity must remain below the maximum permissible exposure (MPE) to ensure safety, though higher laser intensities yield stronger signals, posing risks to the healthy tissues. To enhance PA signals, exogenous contrast agents, including organic dyes, polymers, and metal-based nanoparticles with NIR absorption, are increasingly used. Quality of the output images depends on optical absorption and light fluence in the region of interest (ROI). Further, tissue interference and decreased signal-to-noise ratios while imaging deep seated organs can create irregularities in the received acoustic signals. Additionally, temporal resolution remains a critical issue, with ongoing efforts focused on developing real-time PA imaging systems to improve imaging quality and clinical applicability [12]. Lymphatic imaging, breast cancer, and neurological disorders are established imaging domains of PA systems. Additionally, PAI has also played a pivotal role in image-guided biopsies, targeted drug delivery, and surgical procedures and can be shown in Fig. 1b [13].

Scientific studies utilizing PAI have been showed and effectively testified in the diagnosis of breast cancer, inflammation-mediated ailments such as cancer and rheumatoid arthritis, as well as for the detection of brain tumors [14–16]. In this review, we have elucidated the importance of PAI and PA contrast agents specifically in brain tumor imaging coining the terminology of photoacoustic brain imaging (PABI). Our emphasis has been on using PABI as tool for research and as a medical approach for detecting brain disorders. Utilizing PABI images can offer significant insights into brain disorders and improve existing imaging modalities. Continued investigation could reveal additional practical uses for PABI within the biomedical sector.

Nanophotonics exemplifies the intersection of numerous scientific fields, bringing together ideas from physics, chemistry, material science [17], and biology to transform biomedical imaging [18], specifically in brain imaging. This interdisciplinary collaboration has resulted in the development of novel approaches that use light-matter interactions at the nanoscale [10] to provide non-invasive visualization of complex neural structure. The inclusion of nanomaterials such as quantum dots (QDs) [19], organic/inorganic nanoparticles, and organic dyes [20] into photoacoustic imaging systems has greatly improved imaging resolution and depth, allowing for more precise identification of brain tumors [21] and vascular abnormalities. These materials have exceptional optical characteristics making them ideal for deep tissue penetration and high contrast imaging [22]. The combination of computational modeling [23] and artificial intelligence (AI) expands the potential of nanophotonic brain imaging by addressing the complexity of models [24], processing this data effectively, and recognizing subtle patterns and anomalies indicative of neurological disorder. Simultaneously, computational modeling replicates light while also providing predictive diagnostics. Together, these interdisciplinary technologies enable non-invasive brain imaging with remarkable accuracy, real-time monitoring, and tailored treatment planning, as well as the development of adaptive imaging systems that learn and improve over time, resulting in breakthrough therapeutic innovations.

### Synergistic approaches: photoacoustic agents and ultrasound radiation

Biomedical imaging technologies are vital in modern medicine with respect to timely and precise diagnosis of diseases that guides towards most acceptable treatment plans for enhanced health outcomes [25–27]. Key clinical imaging methods consists of X-ray, CT-scans, MRI, PET scans, optical imaging and US [28, 29]. CT is noted for its high resolution, rapid scanning, and multi-level imaging, but it exposes patients to radiation and has limited tissue differentiation capabilities. MRI provides radiation-free, high-resolution, multi-parametric imaging with excellent soft tissue contrast, but it is time consuming and costly due to its need for exogenous contrast agents and extensive data processing [30]. PET offers molecular-level metabolic insights, useful for early disease detection, treatment evaluation, and tumor diagnosis, but involves radiation exposure and high costs due to radioactive tracers [31]. Optical imaging, with its high contrast and resolution, is extensively used in various medical research fields; however, its imaging depth is limited by photon propagation [32]. Ultrasound imaging, using echo signals from tissue interfaces, enables non-destructive visualization of deeper tissues but struggles with structures smaller than 100 µm without contrast agents, which may reduce sensitivity to disease-related changes. It faces challenges in functional information extraction, like blood oxygenation. Ultrasound’s non-invasive nature is advantageous, though its resolution and target differentiation are lower. The central frequency of ultrasound waves affects penetration depth and resolution balance, critical in applications. Integrating ultrasound with new technologies, particularly photoacoustic imaging, promises non-destructive imaging with improved resolution and penetration compared to pure optical imaging.

In photoacoustic imaging, short laser pulses are directed into tissue, absorbed by biological components such as hemoglobin, causing a sudden rise in temperature followed by thermal expansion, which produces ultrasound waves (PA effect). These waves, carrying structural and compositional information, are detected by ultrasound sensors. Optical imaging is hindered by significant light scattering in biological tissues, restricting imaging to depths of up to 1 mm [32]. Conversely, sound waves scatter 2 to 3 orders of magnitude less than light waves [8–10], permitting deeper tissue penetration, especially in soft tissues [10, 11]. Photoacoustic imaging leverages ultrasound generated by the thermal expansion of heated tissues to capture optical absorption information. Overcoming the penetration depth limitation of optical imaging, enabling imaging up to 7 cm deep [33, 34].

Photoacoustic imaging utilizes the differential absorption characteristics of laser light by different tissues to provide structural and functional information. It facilitated quantitative analysis of tissue components, finely depicting subtle abnormalities and important physiological parameters such as hemoglobin concentration [38], blood oxygenation [39, 40], oxygen metabolism rate [41], blood glucose content [42]. This technology offers cross-scale, multi-functional, non-destructive, and high-resolution biomedical imaging.

**Fig. 2:**
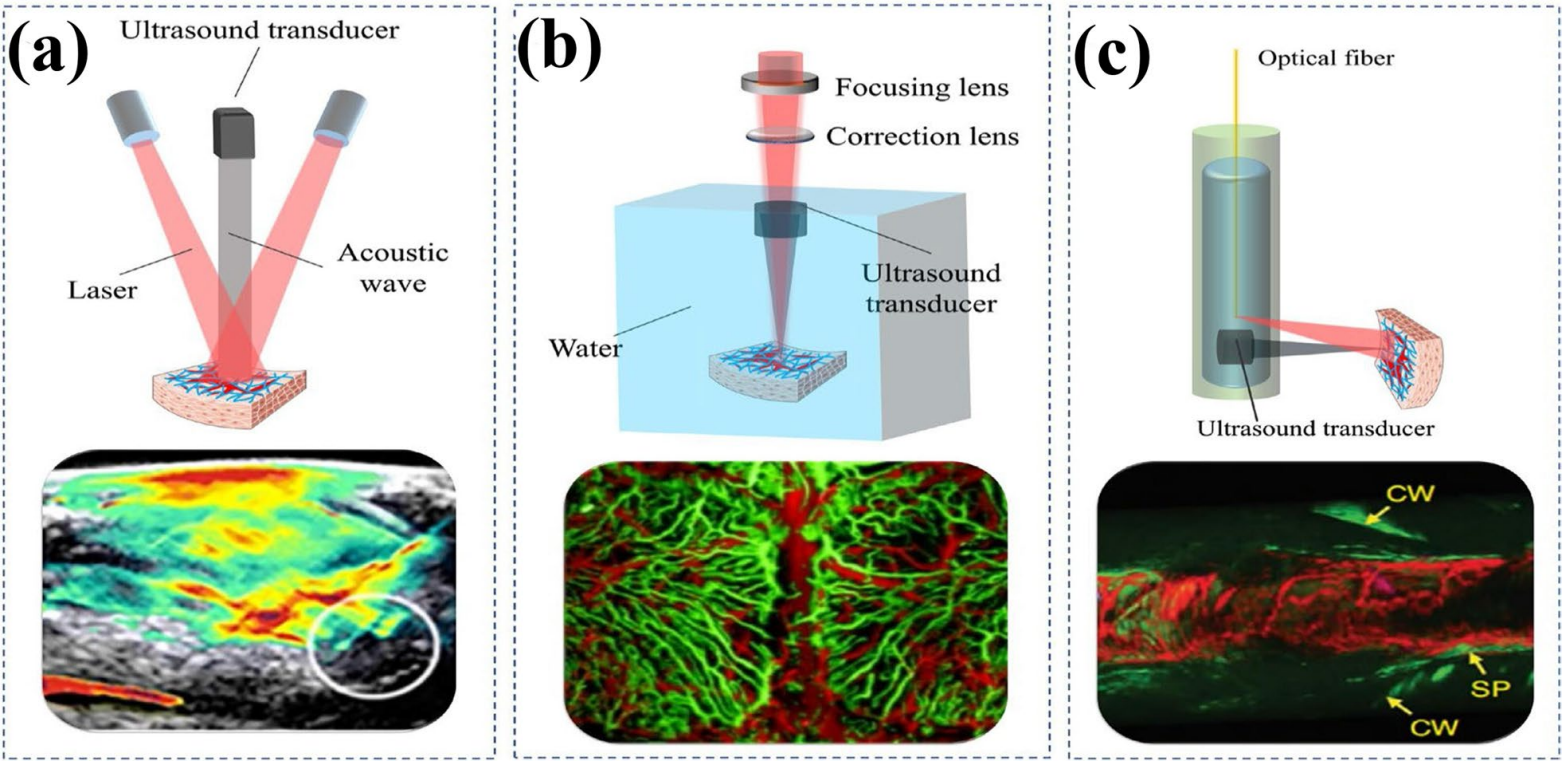
Typical system diagram and imaging results of PAI-US. **a** PACT-US imaging (results from PAI with breast cancer) [35]; **b** PAM-US imaging (results from human brain) [36]; **c** PAE-US imaging (results from a rat colon acquired in vivo) [37]

Photoacoustic imaging is categorized into three types: photoacoustic computed tomography (PACT), photoacoustic microscopy (PAM), and photoacoustic endoscopy (PAE) as shown in Fig. 2a–c. PACT uses full-field illumination with a larger diameter pulsed laser beam for deep tissue and whole-body imaging, providing structural and functional information [43, 44]. PAM employs mechanical scanning with focused ultrasound detectors or laser beams for high-resolution, cellular-level imaging of superficial tissues as presented in Fig. 2b [25, 45]. Photoacoustic endoscopy (PAE) facilitates endoscopic imaging of rat colon is shown in Fig. 2c, useful for evaluating coronary artery diseases, gastrointestinal lesions, and prostate cancer [37].

Combining these techniques with ultrasound imaging (US) overcomes the limitations of individual modalities, achieving high-resolution, high-contrast, deep-penetration, real-time, and non-invasive imaging as presented in Fig. 2a–c. The ultrasound imaging can easily be merged with PACT, PAM, and PAE, detailing their principles, configurations, characterizations, development status, and biomedical applications, while exploring future directions for each approach. The interdisciplinary domains of ultrasound and photoacoustic imaging has played a vital role in biomedical applications specially, to understand the complex structure of brain and still lot of work to be needed for brain imaging due to its vastness and complexity.

According to the detailed discussions, these synergistic approaches of PAI and US imaging have revolutionized biomedical imaging by combining the strengths of each modality. Photoacoustic imaging, encompassing PACT, PAM and PAE, leverages the differential absorption characteristics of laser light to provide detailed structural and functional information across various scales. This includes quantification of critical physiological parameters such as hemoglobin concentration, blood oxygenation, oxygen metabolism rate, and blood glucose content.

PACT excels in deep tissue and whole-body imaging with its full-field illumination, ideal for analyzing large-scale anatomical features, while PAM offers high-resolution cellular-level imaging for superficial tissues. PAE targets specific internal systems like the gastrointestinal tract and coronary arteries. This integration is particularly valuable for brain imaging, where PACT’s deep tissue imaging and PAM’s high resolution provide comprehensive insights into tumor localization, vascularization, and metabolic activity. These combined modalities promise advancements in brain tumor diagnostics and treatment, even more research and advancement are necessary to fully utilize their capabilities. Photoacoustic brain imaging (PABI) uniquely integrates optical and acoustic modalities, providing significant advantages over conventional imaging techniques. It combines the high contrast of optical imaging with the deep tissue penetration of ultrasound, enabling imaging depths of several centimeters—far beyond the superficial reach of fluorescence imaging. PABI achieves high spatial resolution, even at intermediate depths, and supports real-time imaging, making it well-suited for monitoring dynamic physiological changes [46]. Its enhanced contrast arises from the detection of endogenous chromophores (e.g., hemoglobin and melanin) and exogenous contrast agents, facilitating functional and molecular imaging of oxygen saturation, hemoglobin concentration, and vascular structures.

PABI also excels in tumor visualization by delineating clear boundaries and monitoring vascular and metabolic changes in real-time, making it a valuable tool for intraoperative applications [47]. Unlike CT and PET, it employs non-ionizing laser pulses, ensuring safety for repeated and long-term use, while minimizing phototoxicity. The modality supports multispectral imaging to differentiate tissues based on absorption spectra, offering insights into oxygen metabolism and brain hemodynamics. Additionally, PABI systems are cost-effective, portable, and versatile, with applications in neurovascular imaging, neurodegenerative disease research, and drug development, establishing it as a promising tool for diagnosis and prognosis of brain pathologies.

Photoacoustic brain imaging (PABI) offers unique advantages over conventional techniques like MRI, CT, PET, and fluorescence imaging, particularly in tumor boundary depiction, depth detection, and contrast (see Table 1). By integrating optical contrast with acoustic resolution, PABI provides detailed visualization of tumor margins, maps functional and molecular features such as oxygen saturation and hemoglobin concentration, and supports real-time imaging for intraoperative applications. It achieves superior resolution at intermediate depths (up to a few centimeters) and penetrates deeper than fluorescence imaging, while filling the gap between superficial optical methods and whole-brain imaging techniques. PABI also delivers high contrast by exploiting the optical absorption of endogenous chromophores (e.g., hemoglobin, melanin) and exogenous agents, with multispectral imaging providing functional and molecular insights. Unlike CT and PET, it uses non-ionizing radiation, ensuring safety and reducing risks. Additionally, PABI is cost-effective, portable, and capable of dynamic imaging, making it a versatile tool for real-time, high-contrast, and depth-resolved tumor visualization.

**Table 1:** The comparison of relative strengths and weaknesses of PABI with MRI, CT, PET and FL imaging modalities

Modality	Brain tumor location	Vascular imaging	Metabolic activity detection	References
PABI	Strengths: High spatial resolution; optical contrast for clear tumor margins; real-time imaging Weaknesses: Limited depth (few cm); signal attenuation in dense tissues; requires contrast agents	Strengths: Functional imaging of blood flow and oxygenation; high resolution for vascular structures Weaknesses: Resolution decreases with depth; signal attenuation in highly scattering tissues	Strengths: Real-time metabolic changes via hemoglobin oxygenation; multispectral imaging Weaknesses: Indirect metabolic detection; limited by penetration depth	[47–49]
MRI	Strengths: Excellent spatial resolution; 3D imaging; contrast-enhanced MRI improves tumor localization Weaknesses: High cost; bulky infrastructure; not suited for real-time applications	Strengths: Detailed 3D vascular maps via MRA; non-invasive for deep vessels Weaknesses: Limited sensitivity to functional changes in blood flow; expensive and slower imaging	Strengths: Functional MRI detects blood oxygenation and brain activity indirectly. Weaknesses: Slower temporal resolution than PABI; indirect metabolic measurement	[50–52]
CT	Strengths: Rapid imaging; good spatial resolution for calcified tumors Weaknesses: Low contrast for soft tissues; use of ionizing radiation; limited functional insights	Strengths: Accurate imaging of large vessels; rapid imaging capability Weaknesses: Ionizing radiation; lacks sensitivity for small vessels or functional changes	Strengths: Rarely used for metabolic studies but offers structural imaging context Weaknesses: Minimal functional or metabolic information	[53–55]
PET	Strengths: High sensitivity for metabolic activity, aiding tumor localization Weaknesses: Poor spatial resolution; requires radioactive tracers; high cost and complexity	Strengths: Measures perfusion indirectly through metabolic activity Weaknesses: Poor resolution; does not directly visualize vessels	Strengths: Direct metabolic detection via radioactive tracers; highly sensitive functional data Weaknesses: Expensive, requires tracers; limited resolution compared to other techniques	[56–58]
FL Imaging	Strengths: High specificity and sensitivity for surface-level tumors; real-time imaging Weaknesses: Limited penetration depth (< 2 mm); light scattering reduces accuracy in deeper tissues	Strengths: High resolution for superficial vasculature; tracks microvascular changes in real-time Weaknesses: Limited to surface-level vessels; penetration depth restricts applicability	Strengths: Tracks metabolic markers in real-time Weaknesses: Depth-restricted; relies on exogenous markers	[59–61]

## Overview of brain tumors: incidence, diagnosis and treatment approaches

Brain tumors, a diverse collection of cancerous growths in the central nervous system (CNS), are deadly illness with poor survival rates [62]. Over 15,000 cases of primary malignant brain tumors occur each year in the United States [63]. Glioblastoma multiforme (GBM) is the most aggressive type of brain tumor, making up 60% of primary brain cancers and having a low survival rate [64, 65]. GBM is categorized as grade IV by the World Health Organization (WHO), and is the most prevalent and violent type [66]. Despite aggressive standard care treatments, the majority of individuals with brain tumors eventually dies from the illness. Patients with anaplastic astrocytomas have a median survival of about three years, while those with GBM typically survive for around 14.6 months [67, 68].

Brain metastases are notable group of tumors found in the CNS that mainly come from systemic cancers in the lung, breast, and skin [69]. The treatment protocol for malignant brain tumor patients typically includes a combination of surgery, radiation, and chemotherapy [70]. Aggressive tumor resection followed by postoperative radiation has been shown to significantly improve survival rates [71]. Adjuvant chemotherapy can also be given at different times as well [70, 72]. Chemotherapy agents used to treat brain tumors fall into two categories: cytotoxic and cytostatic. They are being operated by the methods like inducing cell death directly, preventing blood vessel formation, blocking growth factor pathways, and hindering tumor invasion. Temozolomide, a derivative of imidazotetrazine, is the primary chemotherapy drug used for patients with brain tumors [73].

**Fig. 3:**
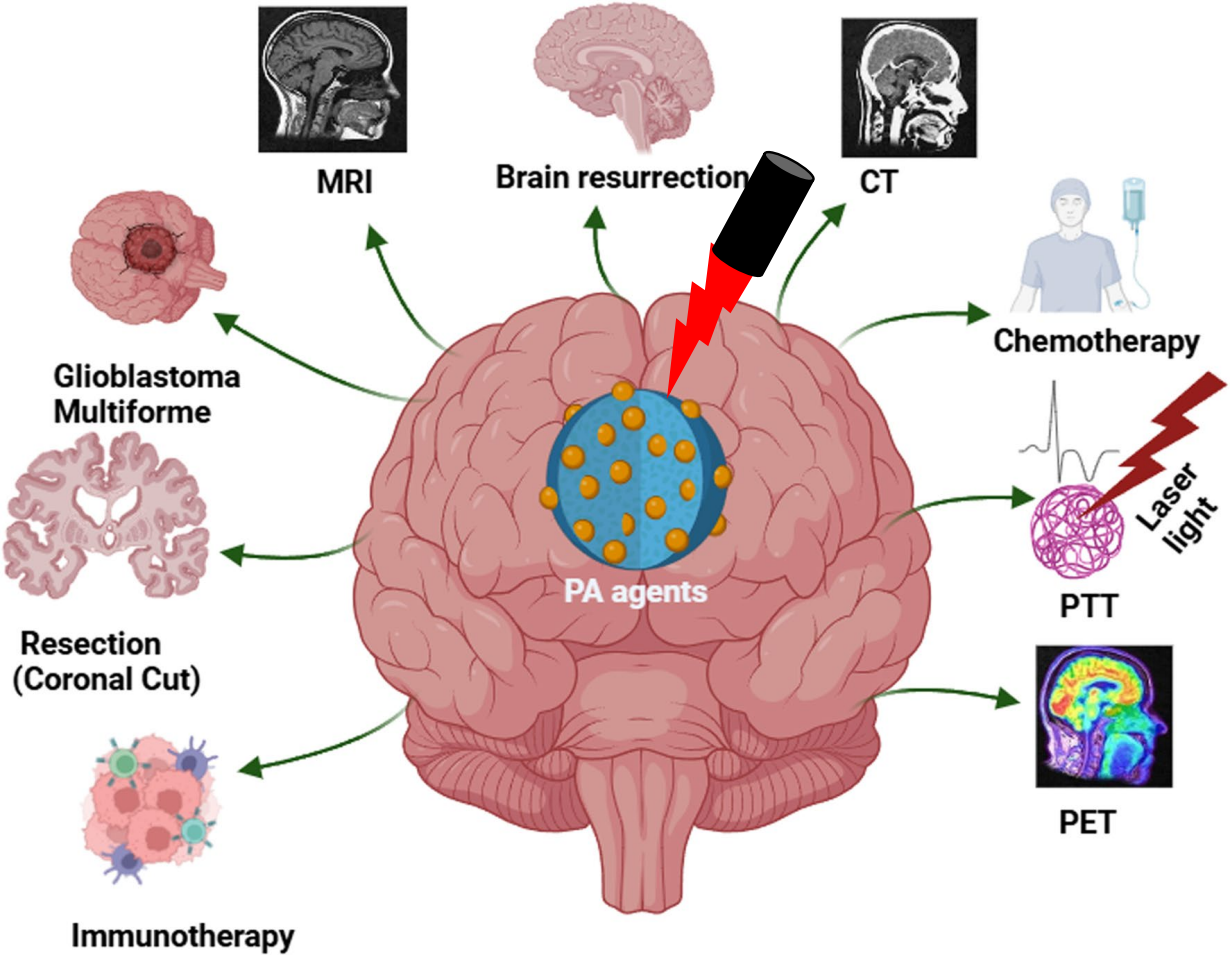
The Schematic illustration with various modalities and treating brain tumors. PET: Positron Emission Therapy, PTT: Photothermal therapy, CT: Computed Tomography, and MRI: Magnetic Resonance Imaging. Created with BioRender.com

Unorthodox treatments like immunotherapy, gene therapy, and photodynamic therapy (PDT) are being tested in clinical trials as supplementary treatments for brain tumors [74, 75]. These new treatments have broadened the selection of therapeutic tools to encompass antibodies, genetic material, and photosensitizers. Moreover, progress in anatomical and functional imaging methods plays a critical role in the treatment of brain tumors by facilitating detection, diagnosis, surgical preparation, and postoperative assessment [76, 77]. The typical imaging methods for diagnosing, elucidating, and imaging brain tumors comprise of MRI, CT, and PET, briefly presented schematically in Fig. 3 [28, 29]. Furthermore, intraoperative fluorescence-guided tumor resection has been improved with the development of fluorescence imaging [78]. Such imaging techniques help identify the borders between cancerous and healthy tissue, helping physicians decide on the best treatment plan.

From the above discussion, GBM is the most severe form of brain cancer, is categorized as grade IV by WHO. Standard treatment involves a multimodal approach; surgical resection, radiotherapy, and chemotherapy. Aggressive resection followed by radiation improves survival, and chemotherapy agents, such as temozolomide, are considered as standard agents. New treatments such as gene therapy, PDT, and immunotherapy are currently being tested in clinical trials. Sophisticated imaging methods are essential for detecting and managing brain tumors effectively diagnosis, surgical planning and follow-up. Key imaging modalities include MRI, CT, PET, and fluorescence imaging for intraoperative guidance. Future directions emphasize enhancing these imaging techniques to better delineate tumor boundaries and integrate novel therapies, ultimately improving treatment outcomes and patient survival.

## Challenges in the treatment of brain tumors

Despite extensive attempts to create diagnostic tools and treatment options, managing brain tumors continuous to be a major trial in the field of neuro-oncology. Key challenges in effectively treating brain tumors include: (a) the intricate structure of the brain; (b) the varied and invasive characteristics of numerous brain tumors; (c) challenges in pinpointing tumor boundaries and spread; (d) inadequate delivery of treatment to the tumor location; and (e) development of resistance to chemotherapy drugs.

The brain, which is possibly the most intricate system in the body, oversees a variety of tasks such as processing information, interpreting sensory input, controlling movement, maintaining balance, generating motivation, acquiring knowledge, and storing memories. Because brain functions are complex, effectively treating brain tumors necessitates the thorough and precise removal of all cancerous tissues, even those that have spread from the main tumor into the surrounding healthy tissue. Experienced surgeons are tasked with successfully identifying all diseased tissue and removing it without damaging the surrounding healthy tissue. Even with thorough removal, brain tumors frequently reappear nearby, typically within a few centimeters of where they were originally removed [66]. Additional therapies, such as chemotherapy, only result in slight improvements in clinical results.

**Fig. 4:**
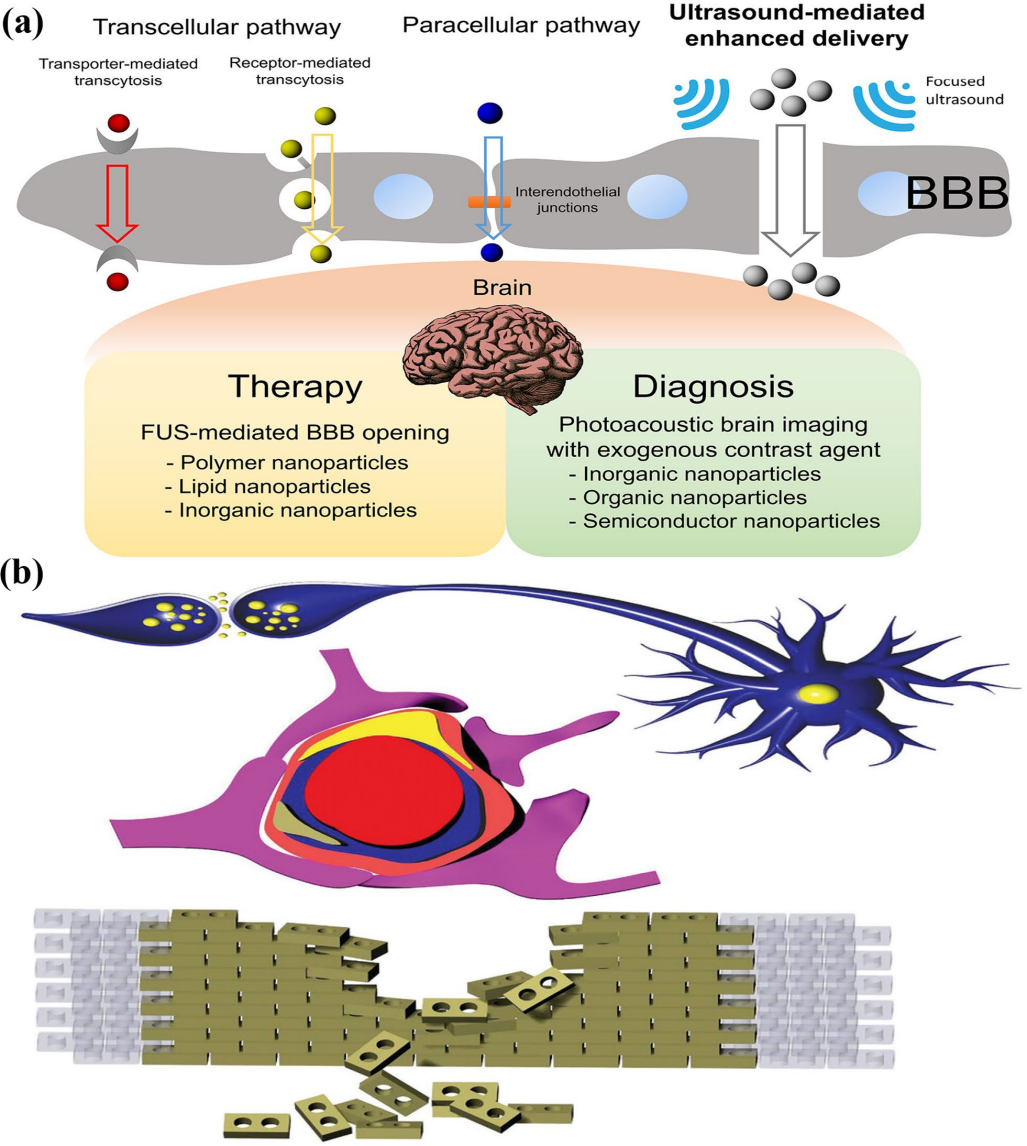
**a** Overview of nanoparticle transport through the blood–brain barrier (BBB) and the applications of ultrasound-mediated nanoparticle delivery in brain research. Inter endothelial junctions form a vascular barrier of the brain that allows the passive diffusion of ions and solutes across the BBB (paracellular pathway). The transcellular pathway includes receptor-mediated transcytosis and transporter proteins that deliver specific molecules through the cytoplasm. The ultrasound-mediated drug delivery method opens up tight junctions between endothelial cells to enhance the permeability of the BBB. These nanoparticles act as therapeutic agents or imaging probes for brain disorders [79]. **b** A schematic illustration of the BBB and its components [81]

Several physiological barriers hinder the effectiveness of systemic delivery of therapeutic agents to brain tumors. In contrast to other body parts, the brain is shielded by the BBB as shown in Fig. 4a, b [79–81]. The BBB stops harmful substances from entering the brain from the blood, while also hindering brain tumor treatment. Numerous studies have focused on overcoming the BBB using nanoparticles. (i) Normally the NPs are transported across the BBB through induced local toxicity which effects the opening of tight junctions between endothelial cells and increase the permeability of the BBB; (ii) receptor-mediated transcytosis that transports molecules through the cytoplasm into the brain; (iii) transporter proteins that deliver specific molecules through endocytosis and exocytosis; or (iv) combination of (i–iii) [80]. Typical brain capillaries function as an unbroken lipid barrier that allows selective passage depending on the solubility and size of molecules. The lack of pinocytotic vesicles in brain endothelial cells also adds to the BBB’s selectivity [80]. Furthermore, ATP-binding cassette carriers, as like P-glycoprotein, function as drug efflux transporters, restricting the transportation of substrate across BBB [82]. Just lipophilic, electro-neutral molecules, and nutrients under 400–600 Da can passively diffuse into the brain [19, 80].

Blood-cerebrospinal fluid (CSF) barrier is the second line of protection which, blocks the passage of systemically administered therapeutic agents [83]. Consisting of closely connected choroid epithelial cells, it controls the entry of molecules into the interstitial fluid of the brain parenchyma. Most large molecules are unable to enter the CSF from the blood due to this obstruction. Additionally, active transportation mechanisms for biological molecules, primarily found in the choroid plexus, reinforce the intact blood-CSF barrier.

The blood-tumor barrier (BTB) in the tumor creates an additional barrier for delivering medical treatments [84]. In contrast to typical brain capillaries, the tight junctions between endothelial cells in the tumor are greatly weakened. The leaky tumor blood vessels cause high pressure inside the tumor, making it difficult for drugs to enter from the bloodstream [85]. Additionally, the existence of various types of tumor micro vessels and variations in capillary functions across the tumor regions results in inconsistence in drug penetration. It might result in heterogenous distribution of drug molecules, significantly compromising therapeutic outcomes.

From above discussion it might be figured out that treating brain tumors remains a formidable challenge due to multiple factors such as the brain’s structural complexity, the heterogenous and invasiveness nature of cancers can make it challenging to determine the boundaries of the tumor, insufficient therapeutic agent accumulation, and acquired drug resistance. The brain’s essential functions necessitate precise elimination of cancerous tissues while preserving surgical resection and adjuvant chemotherapy. Effective drug delivery is hindered by the BBB, CSF barrier, and blood-tumor barrier (BTB), which restrict therapeutic agent penetration. Future treatment directions focus on enhancing imaging techniques such as MRI, CT, PET, and fluorescence imaging to better delineate tumor boundaries, and developing nanoparticle-based therapies to overcome these physiological barriers, ultimately improving diagnosis, surgical precision, and treatment efficacy.

### Influence of PAI in overcoming physiological barriers (BBB/BTB)

Optimal therapeutic outcome can be achieved primarily by enhancing bioavailability of the drug molecules at the disease site and evading the physiological barriers. Blood brain barrier and blood-tumor barrier are the vital factors with which the experts are constantly challenged specifically for the management of brain tumor [86]. Among various external devices that aids the drug formulations to cross the BBB & BTB, PAI has been showing promising outcomes in the recent years. PAI is one of the promising tools for several of cancer diseases which are inaccessible due to several physiological barriers because of its deeper penetrability. Several alternative methodologies separately or in combination, were also employed to improve the theranostic outcome for PAI mediated cancer management [87]. PABI has been reported in several instances where it was instrumental in diagnosis of cancer by providing real-time images with high quality, successful delivery of biomolecular drugs for chemo/gene therapy. The interesting combination of light [9] and sound in this method has open several ventures for usage of multifunctional biomaterials to help in different stages of therapeutic process [88]. The output of PABI can be enhanced by certain contrast agents and these contrast agents formulated along with drug molecules can help in monitoring biodistribution of drugs and PAI guided targeted drug delivery. Drug encapsulated in NPs aided by PAI can help in monitoring drug transport and permeability of BBB/BTB might provide guidance about optimal disruption method [89]. Further, delivery of the drug molecules was also helped by PABI through photothermal or photodynamic mechanisms. Advantages of PAI that involves laser and ultrasound principles have enhanced the multimodal mechanisms, deeper penetration and reduced the invasiveness [90]. PAI also supports multimodal imaging applications by integrating with techniques like PET, MRI, and CT. These combinations provide complementary data, enabling functional imaging of tumor oxygenation, structural mapping of tumor vasculature, and molecular insights into tumor metabolism. This multimodal approach enhances diagnostic accuracy and therapeutic monitoring in brain tumor management.

### Role of PAI in nanoparticles mediated diagnostic imaging and drug delivery

Although, exploiting PAI based methodologies for cancer therapeutic process is still underrated, several nanotechnological and biomaterials-based approaches has shown significant promise in enhancing the diagnostic and therapeutic potential of PABI based devices. Inorganic nanoparticles like gold nanoparticles, iron oxide nanoparticles, and molybdenum disulfide (MoS₂) exhibit strong photoacoustic signals and deep tissue penetration. Gold containing inorganic formulations such as nanospheres, nanocomposites and quantum dots (QDs) were also successful in PABI along with certain hybrid materials such as metal containing nanodroplets. Several nano formulations in the form of organic porphysomes, perfluorocarbon-based nanodroplets, polymeric nanoparticles, were utilized as contrast agents for PABI [91]. Organic nanoparticles, such as ICG-conjugated polymers and novel NIR-II dyes like A1094, improve brain imaging with reduced scattering and enhanced photothermal effects. Semiconductor nanoparticles offer high photothermal conversion efficiency, stability, and effective BBB penetration, making them suitable for brain tumor imaging.

Vascular permeability of tumor was monitored using PAI after intravenous injection of ICG, which could be utilized for screening the targeted delivery of drug molecules [92]. Cu^2+^ based nanomaterial was reported to cross BBB and has shown favor in PABI with Alzheimer’s disease [93]. Similarly, nano formulations made of gold and carbon nanotubes have been studied extensively for crossing BBB, were also utilized for PAI due to the strong NIR light absorption through localized surface plasmon resonance (LSPR) effect [91, 94]. Several organic dye and polymeric nanoparticles were successfully delivered to the tumor site across the tumor vasculature guided by photoacoustic imaging methods. Dendrimers incorporated with organic dyes were delivered across BBB and was successful in imaging and treating glioblastoma using thermal expansion and PA cavitation, respectively [95].

Utilization of gold nanoparticles as a substrate for therapeutic gene containing vectors has shown successful delivery to breast cancer tissue and enhanced treatment outcome [96]. In another study, the silica coated gold nanoparticles incorporated contrast agents have helped successful surgical resection of mouse brain tumor [47]. Utilization of perfluorcarbon nanodroplets containing paclitaxel has shown successful treatment of xenograft tumor in mice through combined photoacoustic-chemotherapy [97]. Liposomal nanoparticles masked by platelet membrane was utilized for NIR II based PAI-guided photothermal therapy for glioma. The study reported effective management of tumor growth in mice with enhanced guidance from PAI [98, 99]. Monitoring drug delivery and therapeutic efficacy was also executed using PAI with support from nanofomulations fabricated with gold nanocages, PLGA based nanoparticles [14], organic dyes like nile blue or methylene blue [100]. Photoacoustic agents have been instrumental in potentiating the PAI based disease management and its theranostic ability against brain tumor has shown remarkable development.

## Fundamental principle of photoacoustic imaging mechanism

The photoacoustic effect depends on varying thermoelastic growth of species when exposed to non-ionizing electromagnetic waves, leading to broad sound generation as explained by Bell in 1880. Research has focused on examining the identification of stress waves caused by lasers in theoretical research [101, 102] cleared the path for photoacoustic microscopes using laser technology [103, 104] and tomographic imaging setups specifically designed for imaging biological tissue [6], as outlined in a recent review [105]. Contemporary biomedical photoacoustic imaging systems commonly employ adjustable laser setups operating in visible/IR spectrum. Tissue chromophores absorb some of the laser pulses, resulting in their transformation into heat and generating a specific area’s temperature rise in millikelvins [106]. The expansion due to change in temperature creates a temporary rise in pressure that travels as wide-ranging acoustic wave through the sample. Ultrasound sensors can detect the wave and use computational methods to create a map of where light-absorbing molecules are located in the tissue [106]. The main factor of image contrast is the diversity in the absorption of laser light within biological substances. Consequently, multispectral laser excitation has the ability to detect particular endogenous/exogenous chromophores by pinpointing their unique photoacoustic spectra [107]. Due to the significantly weaker acoustic scattering compared to optical scattering, PAI can accomplish improved spatial resolution and tissue penetration compared to traditional optical methods. This allows PA imaging to get the abundant data stored in both endogenous/exogenous photo absorbing moieties [46].

### Photoacoustic neuroimaging techniques

**Fig. 5:**
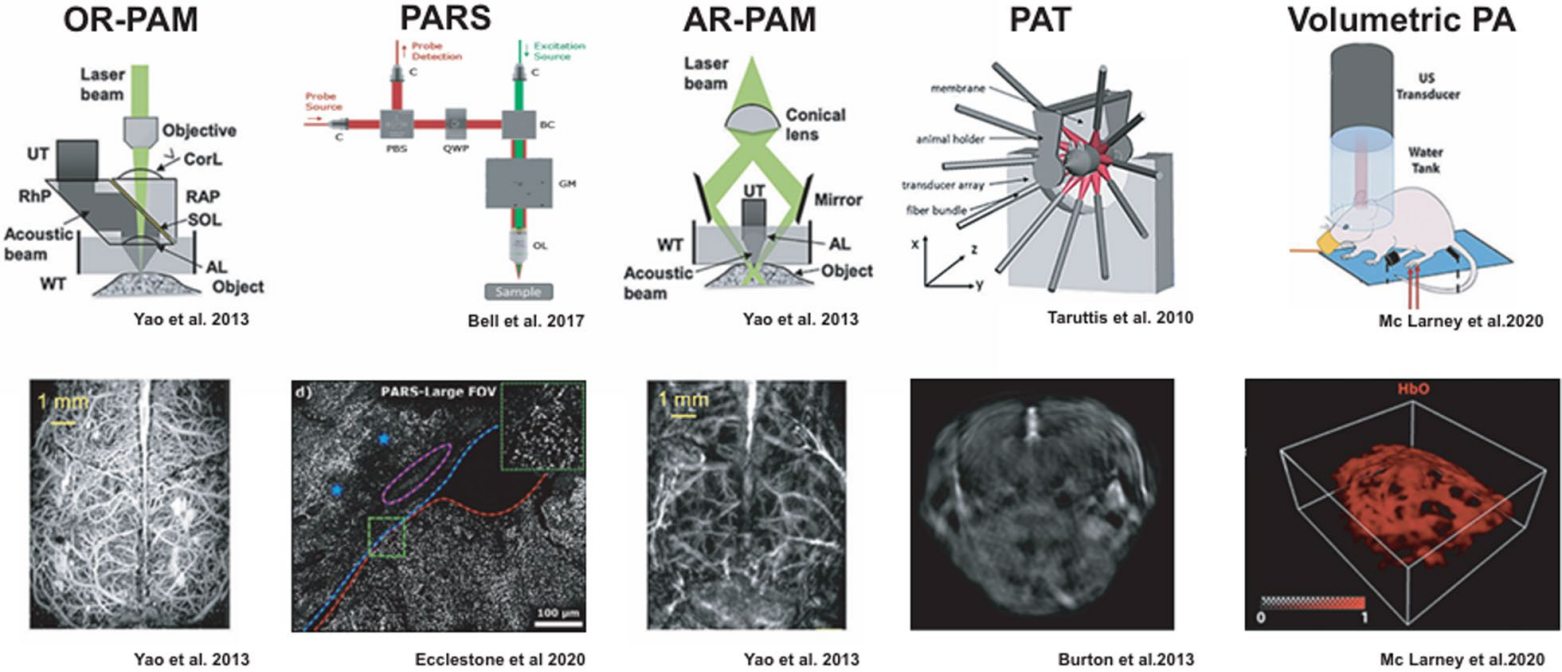
Photoacoustic imaging detection configurations illustrated with neuroimaging outcomes showcased. The image was modified from the cited sources with approval from the authors [110]

In recent years, photoacoustic technology has led to the development of specialized imaging configurations that offer varying trade-offs between spatial and temporal resolution along with depth of penetration [108]. Photoacoustic neuroimaging, a critical use of this technique, combines optical and ultrasonic imaging to generate high-resolution, label-free images of brain structures and functions. It uses the photoacoustic effect, which converts absorbed light into ultrasonic waves, to record precise images of neurovascular networks, functional brain activity, and pathological alterations. Unlike independent imaging techniques, photoacoustic imaging (PAI) provides depth penetration and visual contrast, making it appropriate for non-invasive brain investigations. However, its use in neuroimaging necessitates additional tuning of imaging parameters, data processing methodologies, and contrast agent creation to improve sensitivity and specificity [109]. Experts in the field of photoacoustic imaging have classified the methodologies in order to highlight the practical aspects such as accessibility and their expected outcomes (Fig. 5) [110]. Photoacoustic microscopy (PAM) can be implemented in two ways: by constraining the generation of photoacoustic signals through laser beam focusing, referred to as optical resolution (OR) or by enlightening the sample widely and only capturing ultrasound from a precise focal area, known as acoustic resolution. The process of focusing a laser beam onto a specific area of tissue and collecting the US emitted that focal point using transducer within connecting medium, called optical resolution photoacoustic microscopy (OR-PAM). Resolution is constrained through tiniest optical focal spot size and image visibility is restricted by an optical transport to mean free path of 1 mm [111–114]. Like optical microscopes, laser diffraction is also limited [108, 115]. OR-PAM requires scanning across the sample in a raster pattern and is frequently integrated into integrated microscopes to enhance the visibility of the photo-absorption mapping [115]. PA microscopes have been created due to impracticality of acoustic coupling between the sample and the transducer to remove the necessity for physical connection between the instrument and biological organ. Remote sensing photoacoustic spectroscopy (PARS) tackles this problem by concurrently concentrating a steady probe light in addition to nanosecond stimulation beam. Alteration refers to variations in the brightness of the reflected probe beam in order to measure pressure induced by absorbed light [116, 117]. This setup allows for diffraction-limited resolution without contact at distances of up to 2.5 cm from the tissue being imaged [117], making it appropriate for use during surgery.

On the other hand, AR-PAM works by using laser excitation that covers the whole acoustic identification zone due to lateral resolution being dictated through ultrasonic focusing abilities, (Fig. 5) [118–120]. Image formation occurs by scanning transducers focused on grids and stacking depth profiles from laser shots. AR-PAM applications have the capacity to achieve deep brain imaging up to 5 mm with axial resolutions remaining at 7 µm and lateral resolutions of 30 µm [121]. A demonstration of this is the use of raster-scan optoacoustic mesoscopy (RSOM), which seamlessly integrated with OR-PAM, second, and third harmonic generation microscopy [122].

Instead of moving one transducer through sample, photoacoustic tomography (PAT) utilizes a multiple-element ultrasound sensor arrangement for simultaneous image capture in the sample with a single laser pulse [107, 123–125]. Light is typically transmitted using fiber optic bundles positioned at the edge of the transducer arrangement, and tomographic rebuilding is utilized to calculate 2D images from the PA signals [125, 126]. Either the sample or the transmitter array can be translated to obtain manifold slices. Utilizing quickly adjustable pulsed lasers in the visible/NIR spectrum allows for capturing PA spectra in each sub-volume (MSOT), which enables the mapping of different photo absorber’s distribution using distinct spectra via spectral unmixing methods.

Key methods include photoacoustic microscopy (PAM) and photoacoustic tomography (PAT). PAM has the potential to be executed in the form of optical resolution, known as OR-PAM, which achieves high spatial resolution limited by the optical focal spot size and imaging depth of about 1 mm, or as acoustic resolution (AR-PAM), providing greater imaging depths of up to 5 mm with lateral resolution of 30 µm and axial resolutions of 7 µm. PARS is able to achieve diffraction-limited resolution without making contact up to a distance of 2.5 cm from the tissue, suitable for intraoperative applications. PAT utilizes a multi-element US detector array for simultaneous plane acquisition, facilitating tomographic reconstruction and multi-spectral optoacoustic tomography (MSOT) for mapping photo absorbers. For brain imaging, PARS is particularly promising due to its non-contact and high resolution, though challenges remain in achieving greater penetration depths and improving temporal resolution for real-time imaging.

### Optimization of imaging parameters, data processing and quality assessment in photoacoustic imaging

High quality PA imaging demands careful optimization of imaging parameters, robust data processing methods, and rigorous image quality evaluation standards. These components are critical for improving image performance, ensuring reproducibility and facilitating cross-study comparison. By tailoring these aspects to specific applications, researchers can enhance the reliability and clinical potential of PA imaging.

The adjustment of key imaging parameters is crucial for producing high-quality and reliable PA images. The choice of laser wavelength has a significant impact on imaging performance as it determines the absorption efficiency of target chromophore. NIR wavelengths (700–1300 nm) are preferred for deep tissue imaging due to scattering and greater penetration depth [127]. Pulse duration, typically in millisecond range, is required to cause thermoelastic expansion while avoiding heat accumulation and tissue injury, resulting in significant PA signal under stress confinement conditions [128]. The laser repetition rate ranging from ten to hundreds of Hz, influences imaging speed and temporal resolution, with higher rates allowing real-time monitoring of dynamic processes but needs careful control to avoid tissue photodamage. The energy density must be adequate to generate observable signals while adhering to safety standards, American National Standards Institute (ANSI), limit of 20 mJ/cm^2^ for NIR irradiation. Uniform energy distribution across the field of view is also critical for producing consistent signals and performing correct quantitative analyses. These parameters must be customized to individual applications and meticulously recorded to assure reproducibility, cross-study comparability and safety.

**Fig. 6:**
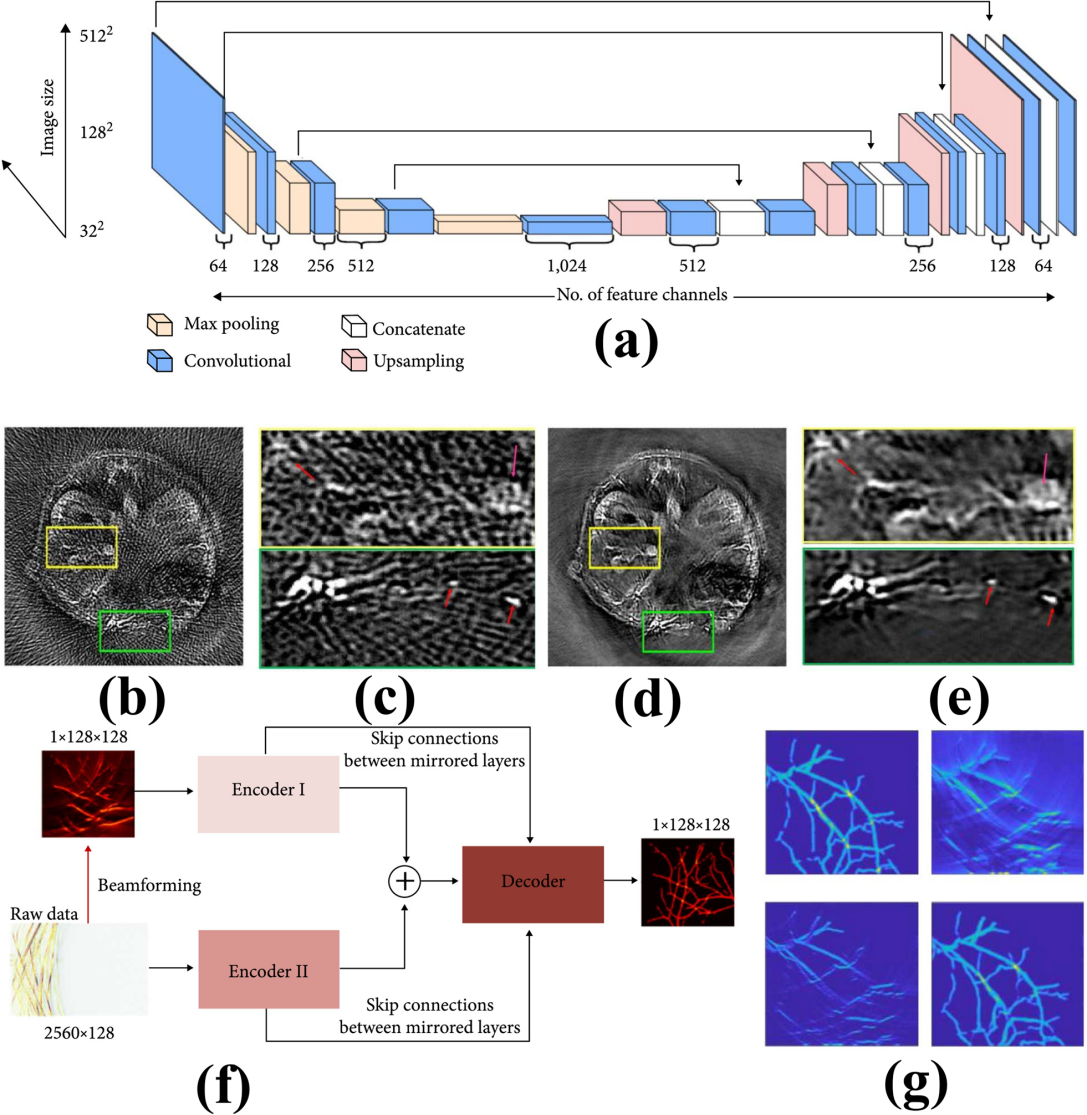
Image reconstructions assisted by machine learning. **a**–**e** Deep learning PAT with sparse data. **a** The U-Net network architecture, consisting of contracting (downsampling) and expansive (upsampling) paths, which is used for the image reconstruction with sparse data. **b** Artifactual reconstructed image with undersampled (128 projections) data, showing the reconstruction artifacts due to the sparse data. **c** Zoom-in images of the yellow and green boxed regions in (**b**). **d** Artifact-free counterpart of (**b**), obtained with the trained network. **e** Zoom-in images of the yellow and green boxed regions in (**d**). **f**, **g** Hybrid neural network for limited-view PACT. **f** The global architecture of Y-Net. Two encoders extract different input features, which concatenate into the decoder. Both encoders have skip connections with the decoder. **g** Comparison of reconstructed images. Top left, ground truth; top right, image reconstructed using the universal back-projection method; bottom left, image reconstructed using the time-reversal method; bottom right, image reconstructed using the trained Y-net [129] with the permission from BME Frontier

Data processing plays a vital role in enhancing the accuracy and quality of PA brain imaging. Preprocessing procedures such as noise reduction, bandpass filtering and signal normalization are critical for reducing artifacts and increasing signal-clarity. Reconstruction algorithms as like delay-and-sum, time-reversal, and model-based approaches are commonly employed to turn raw PA signals into spatially resolved images. Advanced approaches, such as compressed sensing and iterative reconstruction, have increased resolution and processing efficiency. Spectral unmixing techniques are used in multispectral PA imaging to distinguish between chromophores, allowing for functional and molecular imaging. Furthermore, machine learning (ML) and artificial intelligence (AI) techniques are developing as effective methods for automating data interpretation, decreasing noise, and improving image reconstruction as it is illustrated in Fig. 6. The U-Net architecture (Fig. 6a) was trained to detect artifacts during sparse data reconstruction. The trained network was then used to perform in-vivo imaging. Mice were imaged using a PACT scanner with a full ring transducer array of 512 elements (5 MHz central frequency, 80% one-way bandwidth). The reconstructed images with all 512-channel data served as the base for training. The results of artifacts removal from cross-sectional images reconstructed using 128-channel data (illumination wavelength: 1064 nm) are presented in Fig. 6 b-e. The reconstruction algorithm effectively distinguishes small blood vessels from streak artifacts (red arrows in close-up; Fig. 6c, e) and selectively suppresses these artifacts. Here authors have utilized Y-Net, convolutional neural network (CNN) framework, to reconstruct PA images as presented in Fig. 6f by connecting two encoders for texture feature and physical feature. The Y-Net’s performance was tested by numerical simulations in presented in Fig. 6g [129].

Image quality assessment is critical component of PA imaging that guarantees accurate and reliable result interpretation. Image quality is frequently accessed using a number of parameters. The system’s capacity to discriminate between closely spaced structures is determined by its axial and lateral resolution. The acoustic bandwidth affects axial resolution. By measuring the PA signal’s strength in relation to background noise, signal-to-noise ratio (SNR) ensuring that important data is distinguished from artifacts. The contrast-to-noise (CNR) assesses the capacity to distinguish between regions of interest and surrounding tissues, particularly in applications that require the imaging of contrast agent. Another important parameter is quantitative accuracy, especially in research utilizing chromophores or NPs concentration mapping, ensuring a precise link between signal intensity and underlying biological/chemical features. Image quality validation includes comparisons with gold-standard imaging modalities, phantom studies for controlled testing, and real tissue trials to determine biological relevance. Standardized quality assessment standards are critical for reproducibility, allowing for fair comparisons between different systems and studies while also furthering the clinical translation of PA imaging technology.

To ensure cross-compatibility and repeatability in photoacoustic (PA) imaging, similar techniques and practices must be implemented across experimental settings. Key imaging parameters such as laser wavelength, pulse duration, energy density, and repetition rate must be consistently recorded and tailored for the individual application in order to allow replication and comparison across investigations. The use of well-defined phantoms and calibration standards for system validation provides consistency in performance parameters like as resolution, signal-to-noise ratio (SNR), and contrast-to-noise ratio (CNR). The adoption of open-source or standardized data processing algorithms, as well as extensive documenting of pretreatment and reconstruction procedures, improves reproducibility of results. Multicenter partnerships can provide standard protocols, datasets for benchmarking, can help to codify these processes. Finally, making experimental data, raw signals, and reconstruction codes publicly available encourages transparency and allows for independent validation, building confidence and progress in PA imaging research.

To summarize, optimizing imaging parameters, using robust data processing techniques, and establishing stringent quality evaluation standards are critical for enhancing PA imaging. These initiatives will not only improve reproducibility and cross-study comparability, but will also speed up the clinical acceptance of PA imaging, revolutionizing its potential in biomedical research and healthcare applications.

## Principles of photoacoustic and nanophotonic ultrasonic imaging

**Fig. 7:**
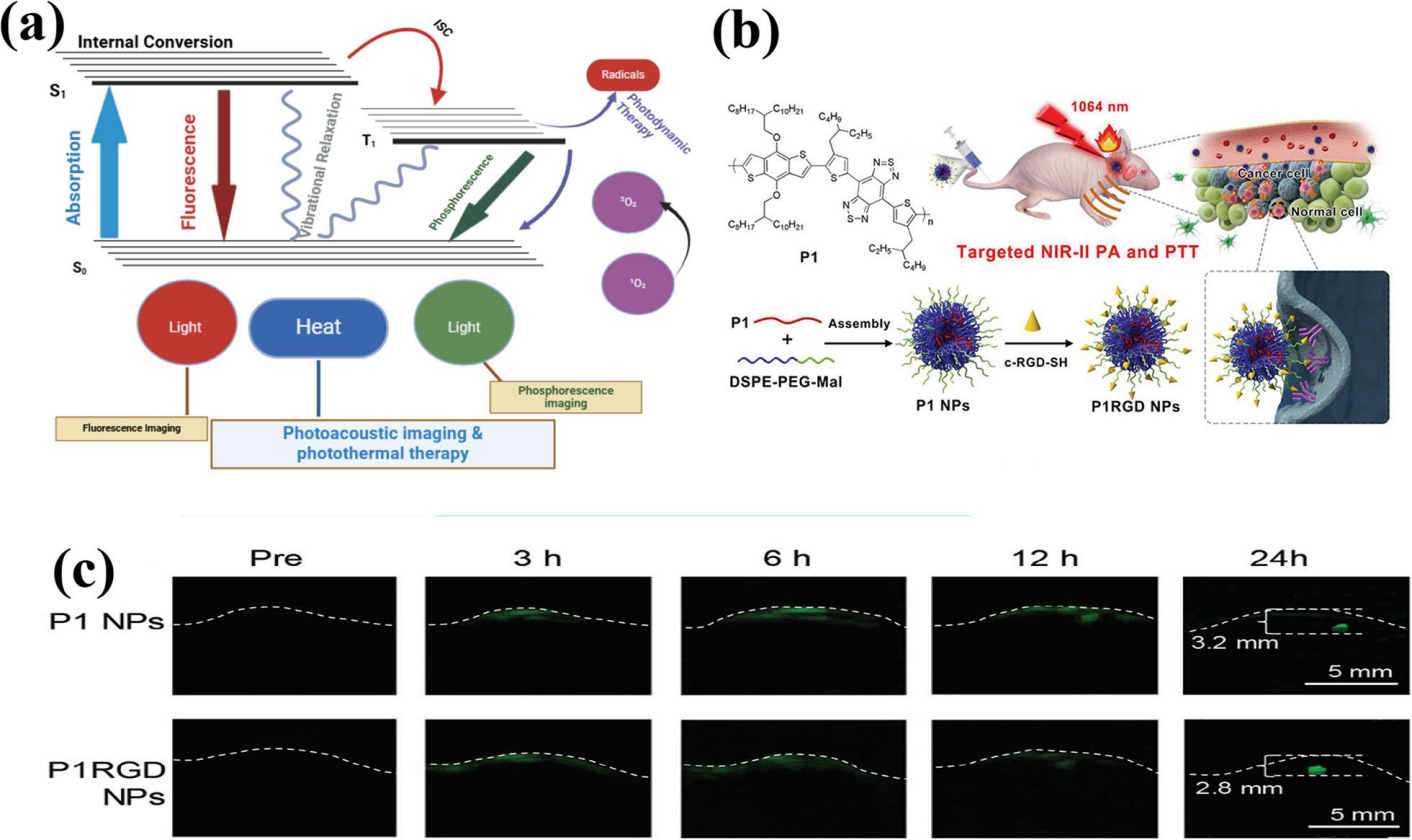
**a** Principles of photoacoustic imaging and photothermal therapy illustrated using a Jablonski diagram [130]. Image was reproduced with permission from the Royal Society of Chemistry and modified using Biorender.com. **b** Schematic diagram of conjugated polymer nanoparticles used for the PA imaging and PTT of brain tumors. **c** images of mouse brain tumor captured with PA technology following the administration of nanoparticles [91]. Image was reproduced with permission from Wiley–VCH Verlag GmbH & Co.

Photoacoustic imaging (PAI) is a cutting-edge, non-invasive practice, employs photon imaging to detect diseases, observe biological tissue structures, and evaluate function. The underlying principle of PAI is the photoacoustic effect in living tissue. When a short-pulsed laser is directed at the imaged sample, the tissue or substance absorbs the light energy, causing thermal elastic expansion and resulting in rapid expansion and contraction of the surrounding medium. Comprehensive fluorescence mechanism of, heat and intersystem conversion is illustrated in the Fig. 7a [130] while the polymer nanoparticles were conjugated with targeting moiety is illustrated in Fig. 7b and allowed for the simultaneous visualization via PA imaging; Fig. 7c [90]. This process generates ultrasound waves that travel towards the tissue surface and are detected. By analyzing the ultrasound signals and utilizing acoustic inverse problems, it is possible to reconstruct the initial sound pressure signal map of the tissue surface. This allows for the observation and diagnosis of biological tissue structure and function [34, 131].

Ultrasound waves and photons differ significantly in scattering intensity within biological tissue (a difference of approximately 2–3 orders of magnitude), resulting in much lower scattering for ultrasound. Consequently, PAI can transcend the diffraction limit of optical imaging depth (1 mm) and offer high-depth, high-contrast, and high-resolution imaging of biological tissue by integrating the imaging depth of ultrasound with the high contrast and resolution of optical imaging.

Photoacoustic imaging (PAI) is a widely used technology with several forms, including photoacoustic tomography (PAT), photoacoustic microscopy (PAM), and photoacoustic endoscopy (PAE) [132]. PAT is a non-invasive imaging technique that uses a large-diameter pulsed laser beam to fully illuminate the tissue surface. This is then followed by the use of an array transducer to collect signals, which are then reconstructed into an image using various inversion algorithms, such as filtered back-projection (FBP), delay-and-sum (DAS) beamforming algorithm, Fourier-based algorithms, and time reversal (TR) algorithm. On the other hand, PAM uses a short-pulsed laser to create a focused point of illumination, followed by the use of a focused transducer to collect the PA signal point-by-point. This eliminates the need for additional inversion algorithms, and allows for image reconstruction. PAE, on the other hand, is an endoscope-based photoacoustic imaging technology that has a wide range of applications in clinical medicine, biomedical research, drug development, material science, and other biomedical fields. Its unique imaging principles and the advantages of optics and acoustics make it a valuable tool in these fields, and its application areas are constantly expanding and deepening.

### Photoacoustic contrast agents for brain tumor imaging

The imaging quality in PAI depends on endogenous factors such as oxyhemoglobin (HbO)/deoxyhemoglobin (Hb), melanin, lipids, and exogenous contrast agents with unique spectral characteristics [133]. Most of the studies conducted before clinical trials on brain imaging have centered around identifying abnormal changes in glioblastoma models, with additional applications in animal models of stroke, epilepsy, Alzheimer’s disease (AD), and neuroinflammation [134–136]. Various kinds of external contrast agents have been established, comprising chemically synthesized dyes/NPs, genetic/semi-genetic agents. Genetically designed Ca^2^^+^ indicators and proteins that can be toggled on and off [137–142]. The requirements for contrast agents in PABI involve having an appropriate absorption (> 600 nm) to distinguish them from endogenic signals of Hb/HbO and melanin, efficient penetration in brain regions, strong attraction with precise binding towards intended goal, effective BBB entry, photostability, solubility, low toxicity, favorable thermodynamics for MRI probes, and ideal pharmacokinetics [133].

Synthetic dyes are often utilized for PA/fluorescence imaging and offer benefits like minimal toxicity, easy passage through BBB because of their small size, quick metabolism, and elimination. Nevertheless, their ability to adapt is restricted. Nanoparticles for imaging consist mainly of carbon, metal, Bi, polymer-incorporated organic constituents, conjugated polymers, and innovative DNA-based nanocarriers [143–149].

NPs provide numerous benefits such as different imaging abilities, a high signal-to-noise ratio, efficient transformation of light into heat, strong ability to reach deeper tissues with NIR-II probes and diverse designs and categories. Nevertheless, careful design is needed to address challenges related to adherence, biodegradability, biocompatibility, minimum toxicity, control over nanostructure, and penetration through BBB [150].

### Chemical dyes for photoacoustic imaging

Numerous chemical dyes commonly employed in PA imaging also exhibit fluorescence characteristics and are frequently used in combined PA/fluorescence imaging [151]. Ideally, PA/fluorescence dyes should have a clear absorption peak and a low quantum yield for effective OA detection. Examples include IR dye 800CW [152], naphthalocyanine, indocyanine green (ICG) [153], and Prussian blue. Administering ICG can visualize blood vessels which allow for PA/fluorescence imaging brain blood flow in a mouse model of glioblastoma [154].

Neuromodulation and activation of glial cells are involved in various brain disorders, like, multiple sclerosis, stroke, and Alzheimer’s disease [155, 156]. Specialized probes, such as those for matrix metalloproteinases (MMPs) and NO generation, used to observe inflammation in nervous system of mice [157]. For example, upregulated MMP levels were detected using an MMP-sense probe (680 nm) with OA/fluorescence imaging in the cerebral ischemic lesion region of a mouse model 48 h after transient middle cerebral artery occlusion [158].

Recent research has revealed the utilization of NIR cyanine derivative CDnir7 for identifying microglia/astroglia initiation in specific regions of brain (triple transgenic AD mice). The buildup and propagation of abnormal proteins play a key role in neurodegenerative disorders. Multiple research studies have utilized PA hybrid dyes that bind to β-sheet structures and have a peak absorbance spectrum in the NIR range for imaging the accumulation of proteopathy in the brain in vivo. Utilizing the oxazine derivative AO1987, PA tomography successfully visualized amyloid-β deposits in mouse models of AD amyloidosis through skull. The same method has been used with an Aβ mouse model, but this time using optical acoustic tomography with the curcumin derivative CRANAD-2 [159]. OA microscopy with Congo red has been utilized to identify amyloid-β plaques and cerebral amyloid angiopathy in the APP/PS1 mouse model [160]. Optical acoustic tomography with the chemical dye PBB5 has been used to detect tau deposits containing β-sheet in the P301L 4-repear (tau mouse) [161].

The OA tomography characteristic ability to detect deep brain regions will likely be utilized with OA/fluorescence β-sheet-binding dyes to image additional proteopathy ailment models like Parkinson’s disease with α-synuclein growth.

According to the discussion it is concluded that chemical dyes (IR dye 800CW, napthalocyanine, ICG, and Prussian) are extensively used in PA imaging due to their dual properties of fluorescence and PA signals, making them ideal for hybrid PA/fluorescence imaging. Among them ICG stands out as a particularly effective dye for PA imaging due to its strong absorption in NIR region, which is ideal for deep tissue imaging. It has the ability to enhance PA imaging of cerebral perfusion highlighting its potential in neuroimaging and provided detailed visualization of vascular structures. The future research should focus on development of new dyes with enhanced sensitivity for different molecular targets associated with brain disorders. These dyes should further proceed to clinical trials to validate their efficacy and safety in humans. The β-sheet-binding dyes might have the potential to image proteopathy related diseases, such as Parkinson’s disease and amyotrophic lateral sclerosis (ALS).

## Photoacoustic brain imaging with nanoparticles

Brain Imaging, crucial for detecting and treating brain diseases, typically utilizes CT, MRI, PET, SPECT [162, 163]. Despite their widespread and high-resolution, is costly and time consuming. CT, PET and SPECT involve ionizing radiation, making them unsuitable for repeated brain measurements [164]. PA imaging offers significant advantages over these traditional modalities, including biosafety, low imaging time, cost-effectiveness, and high spatial resolution [165, 166]. Unlike traditional methods, PA imaging minimizes errors from temporary signals during reconstruction [167] and can be used for vascular patterns and oxygenation mapping via multispectral imaging [168].

**Fig. 8:**
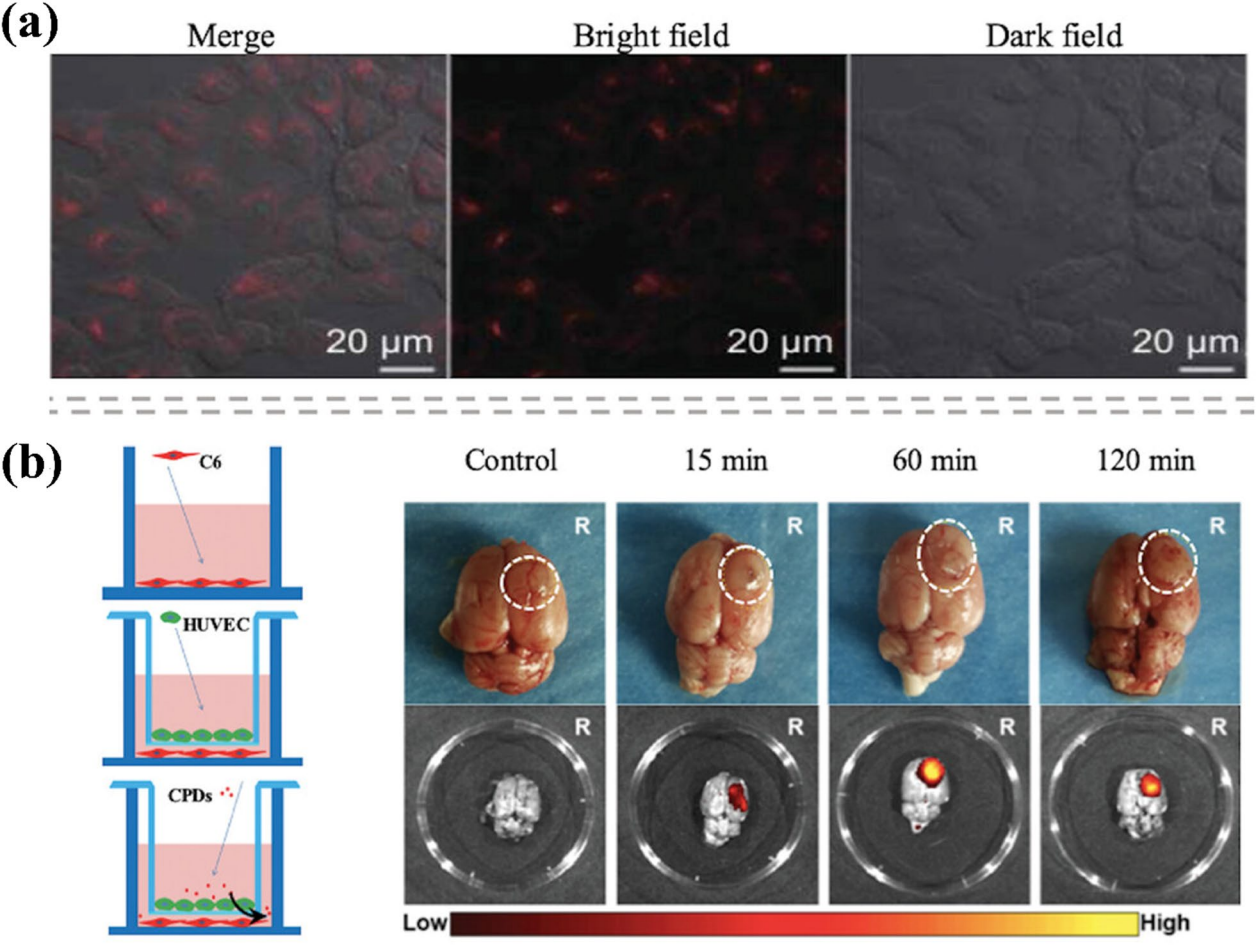
**a** NIR-emitting CDs imaging for HeLa cell [173]. **b** Schematic diagram of the BBB model in vitro, and bright field and fluorescence imaging of NIR-emitting CPDs on the brain of a tumor-bearing mouse ex vivo (white circle shows tumor tissues) [174] taken with permission from the ACS publisher

PA imaging utilizes pulsed laser and light-absorbing contrast agents, generating ultrasound waves through rapid thermal expansion and relaxation of the agents after absorbing light energy. These signals are captured to create PA images. Nanoparticles-based exogenous contrast agents are essential for PA brain imaging due to their unique properties. Nanoparticles absorbing NIR laser enhance the penetration ability of PA imaging, achieving depths of up to 12 cm [169–171]. Notably, a pulsed laser in PA imaging can open BBB with exogenous contrast agents [172]. The small particle size of carbon dots (CDs) allows for their accumulation in tumor regions, facilitating precise imaging. Li et al. [173] employed near-infrared carbon dots (NIR-CDs) with an emission wavelength of 692 nm as imaging probes, co-incubating them with HeLa cells for in vitro imaging studies. The findings demonstrated that NIR-CDs could rapidly penetrate HeLa cells and generate strong NIR emissions localized in the cytoplasm, confirming their excellent cell-labeling capabilities (Fig. 8a). For early diagnosis and visualization of brain tumors, it is crucial that imaging probes possess the ability to cross the blood–brain barrier (BBB). Liu et al. [174] developed carbon-based polymer dots (CPDs) with an emission wavelength of 630 nm and favorable BBB permeability. An in vitro BBB model was constructed using human umbilical vein endothelial cells (HUVEC) and C6 brain glioma cells to evaluate their permeability. In this model, C6 cells were cultured in 12-well plates, while HUVECs were seeded into transwell inserts. CPDs were added to the transwell lumen, and their presence in the fluid of the lower compartment was detected. The fluorescence intensity of CPDs that traversed the BBB model and entered C6 glioma cells served as an indicator of permeability (Fig. 8b). Results revealed that CPDs exhibited a permeability rate of approximately 40% in the in vitro model, highlighting their effective BBB-crossing capabilities. Furthermore, the NIR imaging properties of CPDs enhanced the accuracy of in vivo glioma imaging by minimizing autofluorescence from the skull and scalp. CPDs effectively delineated brain tumor boundaries, demonstrating significant potential for application in surgical visualization and localization of brain tumors.

Furthermore, gold nanoshells have gained attention as a novel contrast-enhancing agent for photoacoustic (PA) tomography. These nanoparticles (NPs) consist of a concentric structure with a dielectric silica core and an outer gold shell. By adjusting the relative thicknesses of the core and shell layers, the optical resonance of gold, derived from its plasmonic properties, can be tuned across a broad wavelength spectrum, ranging from visible to infrared, where physiological transmissivity is maximized. For example, gold nanoshells featuring a 20-nm-thick gold shell and a 100-nm-diameter silica core exhibit an optical absorption peak at 800 nm, enabling in vivo imaging of the vascular architecture in the rat brain [175]. The use of gold nanoshells as contrast agents facilitates precise imaging of the rat brain by significantly enhancing near-infrared contrast in the vasculature. This advancement holds significant potential for biomedical applications, such as in situ tumor detection and tumor therapy guidance based on nanoshell technologies. These findings underscore the capability of gold nanoshells to provide precise, non-invasive imaging at cellular and molecular scales, driven by their efficacy as contrast agents. Additionally, nanoparticle-based PA contrast agents enable simultaneous photothermal therapy (PTT). The effectiveness of PA imaging and PTT is influenced by the photothermal conversion efficiency (PCE), the ratio of generated heat to input radiation energy [20]. Efficient heat generation enhances PA signal amplitude through nonradiative decay. Duan et al. designed NIR-absorbing iron oxide nanoparticles targeting brain tumors for simultaneous imaging and tumor inhibition [176]. Guo et al. developed a PA contrast agent by polymerizing benzodithiophene and benzobisthiadiazole, conjugated with a targeting moiety for dual tumor visualization and treatment [90]. Thus, nanoparticle-based PA imaging is a valuable brain imaging modality. Various nanoparticle agents, including indocyanine green (ICG) [177], carbon nanotubes [178, 179], carbon gold nanoparticles [20, 178], and semiconducting polymer nanoparticles [180, 181], have been developed for visualizing brain tumors and vasculature (see Table 2).

Despite its promise, several barriers limit the widespread adoption of nanoparticle-enhanced PAI. Ensuring biocompatibility and mitigating immune responses or toxicity are critical for clinical translation, particularly for tumor and cardiovascular imaging. Challenges in nanoparticle clearance and retention, including avoiding accumulation in non-target organs, remain a significant hurdle. In neuroimaging, crossing the BBB efficiently and understanding the long-term effects of nanoparticles in brain tissue are technical and safety concerns. Regulatory hurdles complicate the approval process, while the high cost and complexity of nanoparticle synthesis impede scalability. For inflammation imaging and drug delivery, achieving consistent targeting in dynamic environments and integrating imaging with therapeutic functions remain barriers. Furthermore, ensuring nanoparticle stability under physiological conditions and adapting imaging equipment for enhanced PAI functionality increases complexity. Patient-specific responses, including variability in nanoparticle biodistribution and clearance, further complicate standardization. Addressing these challenges through optimized nanoparticle design and system integration can unlock the full potential of PAI in clinical and research settings.

Future developments in PABI contrast agents should focus on designing multifunctional NPs aligning with image and therapeutic application such as drug delivery [182] or PTT and integrating PABI with other modalities like fluorescence, PET, PACT, and MRI for comprehensive diagnosis [31]. Target specific agents with enhanced BBB penetration, achieved through ligand-receptor interactions or US assisted delivery, and molecular targeting for brain-tumor markers or neurodegenerative disease proteins are critical [79]. Advanced chemical dyes absorbing in NIR-II region (1000–1700 nm) for deeper tissue imaging, with improved photostability, can enhance performance. Biodegradable NPs with less toxicity will ensure safety and facilitate clinical translation, complemented by adherence to regulatory standards [183]. Integration with neuroimaging systems using multispectral approaches like MSOT and real-time tracking capabilities will provide dynamic imaging of vascular and metabolic changes. AI-driven innovations, including data-informed NP design and automated analysis, can optimize performance and interpretation. Finally, scalable manufacturing and patient-specific adaptations will support clinical translation, enhancing PA imaging’s potential for personalized and effective diagnostic and therapeutic applications.

### Inorganic/metal nanoparticles for PA brain imaging.

Metal nanoparticles were the pioneering materials identified for their capability to generate robust photoacoustic signals. Among these reported metals, gold NPs have demonstrated superior PA nanoparticles. In this regard, gold nanoparticles are widely utilized as PABI contrast material because of their distinct characteristics such as optical absorption, size, shape and surface functionality. These properties make them highly promising for brain research. Smilowitz et al. found that intravenously injected gold nanoparticles accumulated in intracerebral tumor masses and migrated to tumor cells [184]. The size controllability of gold nanoparticle is particularly advantageous for brain imaging, as it influences their stability to traverse the BBB [185]. Additionally, gold nanoparticle facilitates multimodal brain imaging [186].

**Fig. 9:**
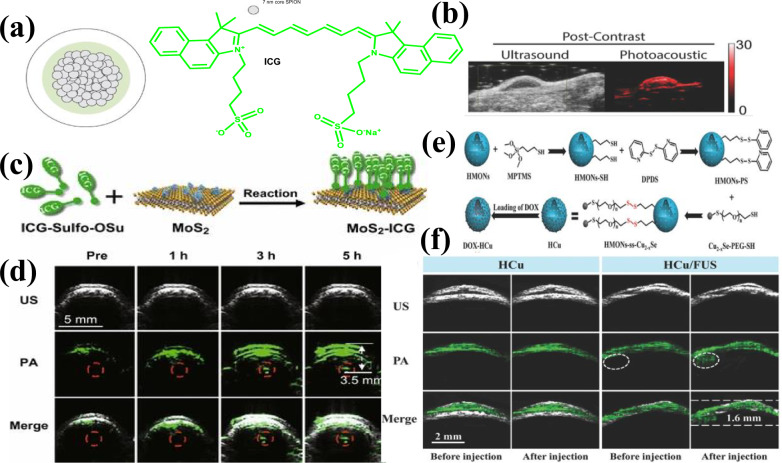
**a** Schematic representation of ICG-iron oxide nanoparticles. **b** US and PA images of U251 xenograft tumors before (left) and 24 h after (right) intravenous injection of a nanoparticle contrast agent. Panels a and b are reproduced from reference [187] with the permission from Wiley–VCH Verlag GmbH & Co. **c** Graphic representation of MoS_2_-ICG hybrid used for PA images of the brain glioma after intravenous injection of MoS_2_-ICG. Red circles indicate the tumor location. **c** and **d** are reproduced from reference [188] with permission from Springer Nature Switzerland AG. **e** Schematic representation of hollow mesoporous organosilica nanoparticles loaded with Cu_2_-xSe NPs and DOX. **f** US, PA, and combined US-PA descriptions showing brain tumors before/after receiving NPs via intravenous injection, with/without BBB opening induced by FUS. **e** and **f** are reproduced from reference [189] with permission of Wiley–VCH Verlag GmbH & Co

Other metals like iron, molybdenum, and copper have also been integrated into nanoparticulate PA agents. Thawani et al. created ICG-coated superparamagnetic nanoparticle for PA brain tumor imaging [187]. These nanoparticles, stabilized with 20–30% ICG, produced strong PA signals in U251-glioma-bearing mice because of better permeability and retention phenomenon. Given both ICG/iron nanoparticles are FDA-approved, clinical applications are promising as shown in Fig. 9a, b.

Chen et al. proposed using MoS_2_ nanosheets for imaging U87 xenograft mouse models [190], leveraging their excellent NIR absorption and PA effect [191]. Improved signals were seen in brain tumors following ICG-conjugated MoS_2_ nanosheets injection. Liu et al. reported that these nanosheets, absorbing NIR light, produced significant PA signals from deep brain gliomas, with signal amplitude 16 times higher than MoS_2_ alone, Fig. 9c, d [188].

After FUS-mediated BBB opening, these DOX-HCu nanoparticles delineated orthotopic brain tumors in PA imaging and selectively release doxorubicin in response to the tumor microenvironment. The study confirmed biodegradability and safety of DOX-HCu, demonstrating its efficacy in brain tumor imaging and therapy. These studies illustrate that PA imaging, augmented with various inorganic nanomaterials, is a potent modality for brain research as presented in Fig. 9e–f [189].

Overall, various type of NPs has been utilized by many researchers with specific advantages such as ICG-coated superparamagnetic iron oxide NPs showed strong PA signals in brain tumors, promising for clinical applications. MoS_2_ nanosheets exhibited excellent NIR absorption, producing enhanced PA signals in brain tumors, while copper-based NPs (DOX-HCu) used with FUS-mediated BBB opening for imaging and therapy with biodegradability and higher efficacy. Among them gold nanoparticles have been identified as highly effective contrast agents for PAI because of their ability to absorb light, size, shape, and surface functionality, which are advantageous for brain research. They accumulate in intracerebral tumor masses and can transverse the BBB, facilitating multimodal brain imaging. Based on this discussion, these NPs should be investigated with respect to safety, efficacy and biodegradability while exploring multimodal imaging. The gold nanoparticles can also be elevated into clinical trials to evaluate their effectiveness and safety in humans.

### Organic nanoparticles for PA brain imaging

Compared to inorganic nanoparticles, organic NPs with NIR absorption provide superior biocompatibility, structural adaptability and optimal optical properties for PA imaging [192]. Consequently, various organic NPs have been developed for brain PA imaging. Organic dyes, notably ICG, are ideal for nanoparticle formulation as PA contrast agents due to their FDA-approved status and NIR absorption capabilities.

Le Floc’h et al. synthesized ICG-conjugated polymer nanoparticles of different sizes (40, 100, 240 nm) to examine size effects on brain delivery. Using flash microprecipitation and FUS-mediated BBB opening post-injection, PA images revealed that 100 and 240 nm nanoparticles were effective for in vivo brain imaging, while 40 nm nanoparticles were not. This discrepancy was attributed to size effects on the NIR absorption ability of ICG nanoparticles, with 100 and 240 nm nanoparticles showing absorbances 5 and 6.3 times greater than 40 nm nanoparticles. The study highlighted the exceptional properties of ICG and identified the optimal nanoparticle size for brain PA imaging.

Liu et al. employed the organic mesionic dye A1094, which absorbs in NIR-II range (1000–1700 nm) exhibiting aggregation-induced absorption enhancement [22]. These properties stem from the π − π conjugation of A1094, resulting in a narrow band gap between the HOMO and LUMO. The NIR-II window (700–1000 nm) offers advantages such as reduced light scattering, lower background signal intensity, and cost efficiency [60]. The authors synthesized Arg-Gly-Asp-modified hepatitis B virus core proteins encapsulating A1094 for PA contrast agent in brain glioma imaging. Post-intravenous injection, the aggregated state of A1094 amplified PA signals in brain tumors 5.9 mm beneath the skull’s surface. These findings demonstrate the suitability of PA organic dye nanoparticles for enhanced brain imaging.

Based on this discussion, ICG-conjugated polymer with larger nanoparticles has enhanced PA effects in brain imaging while novel organic dyes like A1094, which absorb in the NIR-II range, offer advantages such as reduced light scattering and enhanced PA signals, making them suitable for deep penetration leading to deeper tissue imaging. This is due to π–π conjugation resulting in a narrow band gap between HOMO and LUMO leading to strong PA signals. These properties make A1094 a promising choice for advanced PA brain imaging applications. Further research can be carried out to determine the optimal sizes and compositions of the organic nanoparticles for various brain imaging applications.

### Semiconductor nanoparticles for PA brain imaging

**Fig. 10:**
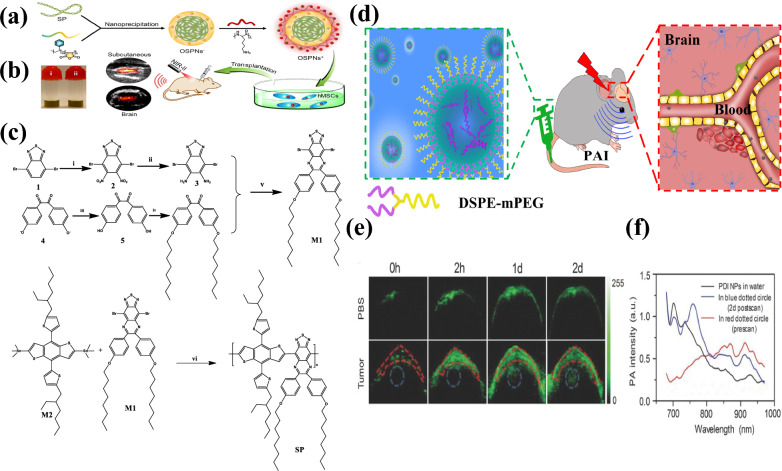
**a** Demonstration of how OSPNs^+^ are prepared and how hMSCs are labeled with PABI after being transplanted. **b** Image showing of the OSPNs^−^ (i) and OSPNs^+^ (ii) solutions. **c** solutions. **c** outline of synthetic process of SP. Conditions of reactions: (i) H_2_SO_4_, HNO_3_ at 100 C; (ii) Fe-powder, CH_3_COOH@80 °C; (iii) pyridine hydrochloride, H_2_O (220–80 °C); (iv) 1-bromooctane, K_2_CO_3_, CH_3_COCH_3_@reflux; (v) CH_3_COOH in N_2_ at 135 °C; (vi) tri-(o-tolyl) phosphine, tris(dibenzylideneacetone) dipalladium in toluene@N_2_ at 100 °C [195]. Image was reproduced with permission from ACS publications. **d** Shows a visual representation of how brain tumors are treated in living organisms using PDI NPs. **e** Coronal brain sections of the control model (top) and tumor model (bottom) were examined before/after injecting 250 µL of 250 nM PDI NPs via tail vain. **f** Spectral analysis of PDI NPs in water solution (black line), highlighted area in red circle in (**e**) before NPs injection (red line) and tumor area in (**e**) after 2 days of NPs injection (blue line) [194]. Images were reproduced with permission from John Wiley and Sons

Semiconducting polymer nanoparticles have emerged as promising contrast agents for PA imaging due to their efficient photothermal conversion [193]. These nanoparticles, utilizing electron donor and acceptor pairs with π-conjugated polymer backbones, exhibit excellent photostability, NIR absorption and enhanced PA effects in brain tumor imaging [181]. Fan et al. established perylene-3,4,9,10-tetracarboxylic diimide (PDI) nanoparticles, featuring a tertiary amine electron donor and a diimide electron acceptor, for deep brain tumor imaging (Fig. 10a) [194]. Modified with DSPE-mPEG 5000 for water stability, these PDI nanoparticles showed enhanced PA signals in animal models with orthotopic glioblastoma upon continuous 700 nm laser irradiation, highlighting their efficacy through EPR effect and low toxicity (Fig. 10b). The synthetic routes for semiconducting polymeric nanoparticles are shown in Fig. 10c along with their reaction conditions [195].

Jiang et al. developed semiconducting polymer nanoparticles with a donor-acceptor1-donor-acceptor2 structure, specifically poly(diketopyrrolopyrrole-alt-thiadiazoloquinoxaline), stabilized in water with a PEG-b-PPG-b-PEG copolymer. These NPs absorbed in both NIR-I and NIR-II regions, improving brain vasculature visualization in rats using a NIR-II excitation laser 156. The 1064 nm laser yielded PA signals 1.4 times more intense than a 750 nm laser. Additionally, the authors created metabolizable NIR-II PA contrast agents using benzobisthiadiazole encapsulated with biodegradable PLGA-PEG, degraded by myeloperoxidase in phagocytes (Fig. 10d). The principle of using DSPE-mPEG NPs as PA agents for brain imaging. The lipids based DSPE-mPEG structure provided stability and functionality. These NPs were intravenously injected to bloodstream in mouse model, where they circulate and reach the brain as shown in Fig. 10d. NIR-II Laser targeted the mouse brain, exciting the NPs to produce US waves (blue ripples), which are detected by PA imaging system. The right panel in Fig. 10d focuses on the brain, showed the interaction of amphiphilic NPs with BBB, crossing into brain tissue, facilitating non-invasiveness imaging of neural structures. This demonstrated the application of DSPE-mPEG NPs in preclinical brain imaging. These NPs visualized brain vasculature effectively, with signal persistence for 3 h post-injection (Fig. 10e), demonstrating their potential for PA brain imaging [14].

Semiconducting polymer nanoparticles (SPNs) have proven to be highly effective contrast agents for PA imaging, particularly in brain tumor imaging. Their unique properties, such as efficient photothermal conversion, excellent photostability, and strong NIR absorption, make them ideal for deep brain imaging. Different donor–acceptor systems have been explored to enhance their PA affects and stability, resulting in nanoparticles that can cross the BBB and provide clear PA signals with low toxicity. The copolymer NPs (pentathiophene donors and benzobisthiadiazole acceptors), Dithieno pyrrole copolymers, and Metabolizable NIR-II PA contrast agents encapsulated with PLGA-PEG showed effective brain vasculature imaging and signal persistence. Among them, PDI NPs stood out for their strong NIR absorption and efficient photothermal conversion. With donor–acceptor feature, these PDI NPs provide robust PA signals, especially when modified with DSPE-mPEG5000. PDI have demonstrated significant PA signal enhancement under 700 nm laser irradiation with orthotopic glioblastoma. These properties make PDI nanoparticles a promising choice for advanced PABI applications.

**Table 2:** Summary of important nanoparticles contrast agents used for photoacoustic brain imaging

Nanoparticle type	Components	Properties	Laser wavelength	Application	Refs.
PDI NPs	PDI/DSPE-mPEG-5000	Enhanced PA signals, water stability	700 nm	Deep brain tumor imaging	[194]
ICG-coated Iron oxide NPs	ICG-SPIO	Strong PA signal FDA-approved	Not specified	Brain tumor imaging	[187]
MoS_2_-ICG hybrid NPs	MoS_2_-ICG (nanosheets)	Enhanced NIR absorption	Not specified	Brain tumor imaging	[188]
DOX-HCu NPS	Copper based NPs, DOX	Biodegradability high efficacy	Not specified	Imaging FUS-mediated BBB opening	[189]
A1094 NPs	Organic mesionic dye Arg-Gly-Asp-modified hepatitis B virus core Proteins	Aggregation-induced absorption enhancement	1000–1700 nm	Deep brain tumor imaging	[22]
PDI NPs	PDI	Efficacy through EPR effect Low toxicity	700 nm	Deep brain tumor imaging	[14]
PLGA-PEG NPs	Benzobisthiadiazole, PLGA-PEG	Biodegradable Strong PA signals	Not specified	Brain vascular visualization	[14]

PDI, perylene-3,4,9,10-tetracarboxylic diimide; ICG, indocyanine green; MoS_2_, molybdenum disulfide; PLGA, poly (lactic-co-glycolic acid); PEG, polyethylene glycol; DOX, doxorubicin

## Future aspects and clinical translation

The review has revealed the significance of nanophotonic enhanced PAI and PA contrast agents particularly for brain tumor imaging. PABI has been the focus of our research efforts and clinical approach for detecting tumor in brain regions. The utility of PA images can provide pertinent information about cerebral pathologies and other conventional imaging modalities [196, 197], there is a possibility of investigating other areas where PAI could be used in future as important tools in biomedical sector.

The synergistic approaches of PAI and US imaging have revolutionized biomedical imaging by combining the strengths of each modality. PAI encompassing PACT, PAM, and PAE, leverages the differential absorption characteristics of laser light to provide detailed structural and functional information across various scales [198]. This includes quantification of critical physiological parameters such as hemoglobin concentration, blood oxygenation [40], oxygen metabolism rate [41], and blood glucose content [42].

PACT is excellent for deep tissue and whole-body imaging using full field illumination, which is most suitable for studying gross anatomical structures, while PAM provides high-resolution cellular level imaging of superficial tissues [199]. On the other hand, PAE focus on particular internal system such as gastrointestinal track and coronary arteries [200]. In particular the use of these two technologies in brain imaging will be beneficial because PACT can help in localizing tumors in deep tissues, it is unable to provide the spatial resolution necessary to assess vascularization or metabolic activity; this resolution can only be solved by means of PAM. These combined modalities have potential applications in enhanced diagnostics and treatment approaches to brain tumor with further research required for them to be fully exploited.

PAI has shown significant progress in delineation of tumor margin and been guiding surgical resection of several type of tumor including the ones originates in deep tissues. The potential of PAI to be utilized as intraoperative imaging technique has shown significant success in tumor removal surgery and several explorations are currently in progress for much affordable and robust process of intraoperative PAI, which would greatly help in surgical procedures pertaining to brain diseases, especially brain tumor. PAI has been employed in detecting residual tumor cells in the lymphatic system by live cell tracking method, plasmonic nanobubbles has been successful in identifying single residual tumor cell at a depth of 4 mm [201]. The success of PAI was also demonstrated in drug delivery research where drug cargos in the form of micro/nano-formulations were successfully accumulated at the TME. Gold nanoparticles have been predominantly successful as PAI mediated drug delivery systems. Monitoring the drug delivery at the disease site was also an important property of PAI, which was demonstrated in small animals. There was a steady increase in number of successful research reported on nanoparticle mediated disease management that involved PAI as a tool for diagnosis, drug delivery, intraoperative imaging, and therapeutic aid, as observed in MRI, CT and other imaging modalities [12].

To treat brain tumor, neuroscientists are involved in multimodal approach; surgical resection, radiotherapy, and chemotherapy. Aggressive resection followed by radiation improves survival, and chemotherapy agents, such as temozolomide [202], are considered as standard agents. Emerging therapies like immunomodulation, gene treatment, and PDT are under clinical trials. Brain tumors management extremely relied on advanced imaging approaches, which help in their detection, diagnosis, surgical planning and follow-up. These include MRI, CT, PET and fluorescence imaging for intraoperative use. The future directions focus on the improvement of these therapies thereby enhancing treatment outcomes and patient survival.

The challenges of treating brain tumors have long been recognized and are due to a number of factors, including: the complexity of brain tissue surrounding it [203], intolerance for movement or physical loss in response to lesions forming around invasive tumors whose margins may be difficult to identify precisely, and the adoption of treatment planning derived from techniques such as radiotherapy and chemotherapy, which sometimes have weak concentrations, leading to poor exposure and eventual resistance. The tissue must be excised to histological margins free of tumors, limiting normal brain involvement during surgical debulking and adjuvant chemotherapy. The blood-tumor barrier (BTB), BCB, and CSF barriers, including the BBB, shield therapeutic agents from effective drug delivery across these barriers [81]. Future treatment directions aim to improve imaging techniques such as MRI, CT, PET, and fluorescence technologies to better define tumor margins, neutralize these physical barriers, and simultaneously enhance nanoparticle-based therapies for better diagnosis guidance, surgical precision, and therapeutic efficacy.

The future of PABI holds significant promise, especially in biomedical applications. A key area of development is the improvement of imaging techniques to overcome physiological barriers (BBB/BTB), which limit penetration of therapeutic agents and pose challenges for effective drug delivery. Future advancements are expected to include the creation of multifunctional nanoparticles (NPs) and the incorporation of sophisticated imaging techniques like CT, PET, MRI, and fluorescence imaging. These innovations aim to enhance the delineation of tumor boundaries, thereby improving the precision of surgical resection and effectiveness of adjuvant therapies. In neuroimaging, PABI holds significant promise for non-invasive imaging. It can produce detailed images of brain structures and functions, aiding in the diagnosis and monitoring of neurological disorders. The ability to visualize vascular networks and blood oxygenation levels is especially useful for understanding disease progression and evaluating therapeutic interventions.

Integrating PAI with other imaging techniques, such as PET and MRI, can significantly improve diagnostic accuracy and treatment planning. For example, PET/CT and PET/MRI provide metabolic and anatomical information, respectively, while PAI contributes functional and molecular imaging capabilities. This multimodal approach enables a more comprehensive and precise diagnosis, facilitating personalized treatment plans and improving patient outcomes.

These photoacoustic neuroimaging techniques have been recently developed with different setups, offering trade-offs between spatial resolution, temporal resolution, and penetration depth. Photoacoustic microscopy (PAM) and photoacoustic tomography (PAT), with their specific advantages, are the main methods used [204]. PAM has been realized as OR-PAM, achieving high spatial resolution constrained by the optical diffraction limit and an imaging depth of about 1 mm. Acoustic resolution PAM (AR-PAM) gains greater imaging depth of approximately 5 mm, with lateral and axial resolutions of around 30 µm and 7 µm, respectively. PARS introduces diffraction-limited resolution up to a few millimeters away in tissue, making it desirable, especially for intraoperative applications. PAT has also become extensively used for both small and large animals in pre-clinical studies, with or without contrast agents, producing 3D quantitative spatially resolved acoustic images. PARS is very promising for brain imaging as it is high-resolution and totally non-contact. Yet, challenges remain in achieving deeper tissue penetration and improving temporal resolution for real-time imaging.

Chemical dyes like IR dye 800 CW, naphthalocyanine organic particle [205], ICG, or Prussian blue can be used to improve PA imaging by providing fluorescence signals. These particles make them suitable for performing hybrid PABI. Among them, ICG is particularly favorable for PABI owing to its high NIR absorption, allowing deep tissue penetration [154]. This development significantly strengthens cerebral perfusion detection in PABI and opens up new prospects for neuroimaging by providing a detailed view of vasculature structures. Future research should focus on developing novel dyes for more specific brain disorder-associated targets. These dyes, currently studied in pre-clinical research, must undergo clinical testing to verify their efficacy and safety. For example, β-sheet-binding dyes [206] could serve as useful probes to image proteinopathy-related diseases such as Parkinson’s and amyotrophic lateral sclerosis (ALS).

Besides chemical dyes, various types of nanophotonic based nanoparticles have been implemented to enhance PABI. ICG-coated superparamagnetic iron oxide NPS have demonstrated powerful PA signal in brain tumors mediated by enhanced NIR absorption of MoS_2_ nanosheets, while copper-based NPs, DOX-HCu, have been employed for imaging under focused ultrasound (FUS)-triggered BBB opening, offering biodegradability with high effectiveness. AuNPs are particularly desirable as contrast agents in PA imaging of the brain due to their biocompatible optical absorption and essential characteristic like size, shape compatibility, and surface functionality. These NPs aggregate in intracerebral tumor masses and can cross BBB, facilitating multimodal brain imaging. Given these positive outcomes, further research is needed to confirm their safety and efficacy in humans as the next steps toward clinical trials.

Thus, starting from NPs, especially CDs and expanding further, the newest modifications of the ICG-conjugated polymers have increased nanoparticle size providing to improve PA effects in brain imaging. At the same time, new organic dyes like A1094 under NIR-II excitation also provided such benefits as minimized scattering and increased PA signals. These features make them particularly suitable for deep tissue imaging due to the π–π conjugation in the dye’s molecular structure, which translates into a low energy gap between the highest occupied molecular orbital (HOMO) and the lowest unoccupied molecular orbital (LUMO) [207], thus providing intense PA signals. Therefore, through the described properties of A1094 it can be offered as a candidate for advanced PABI. Thus, further studies can be carried out on finding out the extent of potentiality of these organic NPs for various needs of neuroimaging which can be extended to the development of better imaging methods with increased accuracy and penetration.

Furthermore, machine learning (ML) and artificial intelligence (AI) can dramatically improve image quality, data processing, and reconstruction in photoacoustic brain imaging (PABI). AI-powered algorithms can reduce noise in raw photoacoustic signals, improve resolution, and optimize contrast, resulting in clearer and more detailed photos. Advanced reconstruction methods, such as deep learning-based neural networks, can speed up image reconstruction while maintaining spatial accuracy and reducing artifacts. Furthermore, ML models can simplify data processing workflows, eliminate human bias, and detect subtle patterns, hence increasing the overall reliability and repeatability of photoacoustic imaging for brain applications.

## Conclusion

Photoacoustic imaging represents a significant advancement in biomedical imaging, offering unique capabilities that traditional imaging modalities lack. Its capability to deliver high-resolution, real-time images containing functional and molecular information enable it an invaluable tool in clinical practice. The future of PABI lies in its continued development and integration with other imaging techniques, overcoming physiological barriers, and enhancing its clinical applications. Advancements in nanoparticles-based therapies and imaging techniques will likely improve the precision of diagnosis and the efficacy of adjuvant therapies. The evolution of neuroimaging techniques will offer better spatial and temporal resolution, aiding in management of neurological diseases. The clinical translation of these technologies will revolutionize the early detection, diagnosis, and treatment of various diseases, particularly cancer and neurological disorders.

In conclusion, photoacoustic imaging holds immense potential for improving patient outcomes through its innovative approach to biomedical imaging. Continued research and development in, spanning from contrast agents through various modalities to AI assisted techniques, this area will lead to improved outcomes and personalized medical treatments, ultimately enhancing the quality of healthcare.

## Data Availability

No datasets were generated or analysed during the current study.

## References

[1] Shen Y, Prasad PN. Nanophotonics: a new multidisciplinary frontier. Appl Phys B. 2002;74:641–5.

[2] Iqbal MA, Malik M, Shahid W, Ahmad W, Min-Dianey KA, Pham PV. Plasmonic 2D materials: overview, advancements, future prospects and functional applications. In: Pham PV, editor. 21st Century nanostructured materials: physics, chemistry, classification, and emerging applications in industry, biomedicine, and agriculture. London: IntechOpen; 2021. p. 47–68.

[3] Malik M, Iqbal MA, Malik M, Raza MA, Shahid W, Choi JR, Pham PV. Biosynthesis and characterizations of silver nanoparticles from *Annona**squamosa* leaf and fruit extracts for size-dependent biomedical applications. Nanomater. 2022;12:616. 10.3390/nano12040616 PMC8879340 35214945

[4] Conde J, Rosa J, Lima JC, Baptista PV. Nanophotonics for molecular diagnostics and therapy applications. Int J Photoenergy. 2012;2012: 619530.

[5] Malik M, Aamir Iqbal M, Iqbal Y, Malik M, Bakhsh S, Irfan S, Ahmad R, Pham PV. Biosynthesis of silver nanoparticles for biomedical applications: a mini review. Inorg Chem Commun. 2022;145: 109980.

[6] Wang X, Pang Y, Ku G, Xie X, Stoica G, Wang LV. Noninvasive laser-induced photoacoustic tomography for structural and functional in vivo imaging of the brain. Nat Biotechnol. 2003;21:803–6. 12808463 10.1038/nbt839

[7] Wang LV, Hu S. Photoacoustic tomography: in vivo imaging from organelles to organs. Science. 2012;335:1458–62. 22442475 10.1126/science.1216210 PMC3322413

[8] Manohar S, van Apeldoorn A, Steenbergen W. Cells make themselves heard. Nat Photonics. 2015;9:216–8.

[9] Wang LV. Prospects of photoacoustic tomography. Med Phys. 2008;35:5758–67. 19175133 10.1118/1.3013698 PMC2647010

[10] John S, Hester S, Basij M, Paul A, Xavierselvan M, Mehrmohammadi M, Mallidi S. Niche preclinical and clinical applications of photoacoustic imaging with endogenous contrast. Photoacoustics. 2023;32: 100533. 37636547 10.1016/j.pacs.2023.100533 PMC10448345

[11] Chen L, Wu Y, Zhang W, Shen W, Song J. Imaging-guided antibacterial based on gold nanocrystals and assemblies. Small Methods. 2024;8:2301165. 10.1002/smtd.202301165 37798919

[12] Sridharan B, Lim HG. Advances in photoacoustic imaging aided by nano contrast agents: special focus on role of lymphatic system imaging for cancer theranostics. J Nanobiotechnology. 2023;21:437. 37986071 10.1186/s12951-023-02192-8 PMC10662568

[13] Steinberg I, Huland DM, Vermesh O, Frostig HE, Tummers WS, Gambhir SS. Photoacoustic clinical imaging. Photoacoustics. 2019;14:77–98. 31293884 10.1016/j.pacs.2019.05.001 PMC6595011

[14] Fan Q, Cheng K, Yang Z, Zhang R, Yang M, Hu X, Ma X, Bu L, Lu X, Xiong X, et al. Perylene-diimide-based nanoparticles as highly efficient photoacoustic agents for deep brain tumor imaging in living mice. Adv Mater. 2015;27:843–7. 25376906 10.1002/adma.201402972 PMC4347809

[15] Oraevsky A, Clingman B, Zalev J, Stavros A, Yang W, Parikh J. Clinical optoacoustic imaging combined with ultrasound for coregistered functional and anatomical mapping of breast tumors. Photoacoustics. 2018;12:30–45. 30306043 10.1016/j.pacs.2018.08.003 PMC6172480

[16] Jo J, Tian C, Xu G, Sarazin J, Schiopu E, Gandikota G, Wang X. Photoacoustic tomography for human musculoskeletal imaging and inflammatory arthritis detection. Photoacoustics. 2018;12:82–9. 30596016 10.1016/j.pacs.2018.07.004 PMC6306364

[17] Shen Y, Friend CS, Jiang Y, Jakubczyk D, Swiatkiewicz J, Prasad PN. Nanophotonics: interactions, materials, and applications. J Phys Chem B. 2000;104:7577–87.

[18] Im S, Mousavi S, Chen Y-S, Zhao Y. Perspectives of chiral nanophotonics: from mechanisms to biomedical applications. NPJ Nanophotonics. 2024;1:46.

[19] Zhou Y, Peng Z, Seven ES, Leblanc RM. Crossing the blood-brain barrier with nanoparticles. J Control Release. 2018;270:290–303. 29269142 10.1016/j.jconrel.2017.12.015

[20] Yang M, Ji C, Yin M. Aggregation-enhanced photothermal therapy of organic dyes. Wiley Interdiscip Rev Nanomed Nanobiotechnol. 2024;16: e1960. 38695260 10.1002/wnan.1960

[21] Zhao W, Yu X, Peng S, Luo Y, Li J, Lu L. Construction of nanomaterials as contrast agents or probes for glioma imaging. J Nanobiotechnology. 2021;19:125. 33941206 10.1186/s12951-021-00866-9 PMC8091158

[22] Liu Y, Liu H, Yan H, Liu Y, Zhang J, Shan W, Lai P, Li H, Ren L, Li Z, Nie L. Aggregation-induced absorption enhancement for deep near-infrared II photoacoustic imaging of brain gliomas in vivo. Adv Sci. 2019;6:1801615. 10.1002/advs.201801615 PMC6469237 31016108

[23] Winge DO, Limpert S, Linke H, Borgström MT, Webb B, Heinze S, Mikkelsen A. Implementing an insect brain computational circuit using III–V nanowire components in a single shared waveguide optical network. ACS Photonics. 2020;7:2787–98. 33123615 10.1021/acsphotonics.0c01003 PMC7587142

[24] Kotwal A, Saragadam V, Bernstock JD, Sandoval A, Veeraraghavan A, Valdés PA. Hyperspectral imaging in neurosurgery: a review of systems, computational methods, and clinical applications. J Biomed Opt. 2025;30:023512–023512. 39544341 10.1117/1.JBO.30.2.023512 PMC11559659

[25] Yu Y, Feng T, Qiu H, Gu Y, Chen Q, Zuo C, Ma H. Simultaneous photoacoustic and ultrasound imaging: a review. Ultrasonics. 2024;139: 107277. 38460216 10.1016/j.ultras.2024.107277

[26] Richards-Kortum R, Lorenzoni C, Bagnato VS, Schmeler K. Optical imaging for screening and early cancer diagnosis in low-resource settings. Nat Rev Bioeng. 2024;2:25–43. 39301200 10.1038/s44222-023-00135-4 PMC11412616

[27] Acosta JN, Falcone GJ, Rajpurkar P, Topol EJ. Multimodal biomedical AI. Nat Med. 2022;28:1773–84. 36109635 10.1038/s41591-022-01981-2

[28] Martucci M, Russo R, Schimperna F, D’Apolito G, Panfili M, Grimaldi A, Perna A, Ferranti AM, Varcasia G, Giordano C, Gaudino S. Magnetic resonance imaging of primary adult brain tumors: state of the art and future perspectives. Biomedicines. 2023;11:364. 36830900 10.3390/biomedicines11020364 PMC9953338

[29] Bin-Alamer O, Abou-Al-Shaar H, Gersey ZC, Huq S, Kallos JA, McCarthy DJ, Head JR, Andrews E, Zhang X, Hadjipanayis CG. Intraoperative imaging and optical visualization techniques for brain tumor resection: a narrative review. Cancers (Basel). 2023;15:4890. 37835584 10.3390/cancers15194890 PMC10571802

[30] Chaban YV, Vosshenrich J, McKee H, Gunasekaran S, Brown MJ, Atalay MK, Heye T, Markl M, Woolen SA, Simonetti OP, Hanneman K. Environmental sustainability and MRI: challenges, opportunities, and a call for action. JMRI. 2024;59:1149–67. 37694980 10.1002/jmri.28994 PMC11707703

[31] Robson N, Thekkinkattil DK. Current role and future prospects of positron emission tomography (PET)/computed tomography (CT) in the management of breast cancer. Med. 2024;60:321. 10.3390/medicina60020321 PMC10889944 38399608

[32] Yoon S, Cheon SY, Park S, Lee D, Lee Y, Han S, Kim M, Koo H. Recent advances in optical imaging through deep tissue: imaging probes and techniques. Biomater Res. 2022;26:57. 36273205 10.1186/s40824-022-00303-4 PMC9587606

[33] Riksen JJM, Nikolaev AV, van Soest G. Photoacoustic imaging on its way toward clinical utility: a tutorial review focusing on practical application in medicine. J Biomed Opt. 2023;28: 121205. 37304059 10.1117/1.JBO.28.12.121205 PMC10249868

[34] Jiang D, Zhu L, Tong S, Shen Y, Gao F, Gao F. Photoacoustic imaging plus X: a review. J Biomed Opt. 2024;29:S11513–S11513. 38156064 10.1117/1.JBO.29.S1.S11513 PMC10753847

[35] MacCuaig WM, Jones MA, Abeyakoon O, McNally LR. Development of multispectral optoacoustic tomography as a clinically translatable modality for cancer imaging. Radiol Imaging Cancer. 2020;2:e200066. 33330850 10.1148/rycan.2020200066 PMC7706874

[36] Estrada H, Rebling J, Hofmann U, Razansky D. Discerning calvarian microvascular networks by combined optoacoustic ultrasound microscopy. Photoacoustics. 2020;19:100178. 32215252 10.1016/j.pacs.2020.100178 PMC7090363

[37] Yang J-M, Favazza C, Chen R, Yao J, Cai X, Maslov K, Zhou Q, Shung KK, Wang LV. Simultaneous functional photoacoustic and ultrasonic endoscopy of internal organs in vivo. Nat Med. 2012;18:1297–302. 22797808 10.1038/nm.2823 PMC3885361

[38] Hacker L, Brunker J, Smith ESJ, Quiros-Gonzalez I, Bohndiek SE. Photoacoustics resolves species-specific differences in hemoglobin concentration and oxygenation. J Biomed Opt. 2020;25: 095002. 32888263 10.1117/1.JBO.25.9.095002 PMC7471783

[39] Li M, Tang Y, Yao J. Photoacoustic tomography of blood oxygenation: a mini review. Photoacoustics. 2018;10:65–73. 29988848 10.1016/j.pacs.2018.05.001 PMC6033062

[40] Karmacharya MB, Sultan LR, Sehgal CM. Photoacoustic monitoring of oxygenation changes induced by therapeutic ultrasound in murine hepatocellular carcinoma. Sci Rep. 2021;11:4100. 33603035 10.1038/s41598-021-83439-y PMC7893035

[41] Sun N, Bruce AC, Ning B, Cao R, Wang Y, Zhong F, Peirce SM, Hu S. Photoacoustic microscopy of vascular adaptation and tissue oxygen metabolism during cutaneous wound healing. Biomed Opt Express. 2022;13:2695–706. 35774317 10.1364/BOE.456198 PMC9203110

[42] Jin Y, Yin Y, Li C, Liu H, Shi J. Non-invasive monitoring of human health by photoacoustic spectroscopy. Sensors (Basel). 2022;22:1155. 35161900 10.3390/s22031155 PMC8839463

[43] Attia ABE, Balasundaram G, Moothanchery M, Dinish US, Bi R, Ntziachristos V, Olivo M. A review of clinical photoacoustic imaging: current and future trends. Photoacoustics. 2019;16: 100144. 31871888 10.1016/j.pacs.2019.100144 PMC6911900

[44] Yao J, Wang LV. Photoacoustic tomography: fundamentals, advances and prospects. CMMI. 2011;6:332–45. 22025335 10.1002/cmmi.443 PMC3205414

[45] Liu W, Yao J. Photoacoustic microscopy: principles and biomedical applications. Biomed Eng Lett. 2018;8:203–13. 30603203 10.1007/s13534-018-0067-2 PMC6208522

[46] Ntziachristos V, Razansky D. Molecular imaging by means of multispectral optoacoustic tomography (MSOT). Chem Rev. 2010;110:2783–94. 20387910 10.1021/cr9002566

[47] Kircher MF, de la Zerda A, Jokerst JV, Zavaleta CL, Kempen PJ, Mittra E, Pitter K, Huang R, Campos C, Habte F, et al. A brain tumor molecular imaging strategy using a new triple-modality MRI-photoacoustic-Raman nanoparticle. Nat Med. 2012;18:829–34. 22504484 10.1038/nm.2721 PMC3422133

[48] Yao J, Wang L. Photoacoustic brain imaging: from microscopic to macroscopic scales. Neurophotonics. 2014;1: 011003. 25401121 10.1117/1.NPh.1.1.011003 PMC4232215

[49] Zhu X, Huang Q, DiSpirito A, Vu T, Rong Q, Peng X, Sheng H, Shen X, Zhou Q, Jiang L, et al. Real-time whole-brain imaging of hemodynamics and oxygenation at micro-vessel resolution with ultrafast wide-field photoacoustic microscopy. Light Sci Appl. 2022;11:138. 35577780 10.1038/s41377-022-00836-2 PMC9110749

[50] Majos A, Tybor K, Stefańczyk L, Góraj B. Cortical mapping by functional magnetic resonance imaging in patients with brain tumors. Eur Radiol. 2005;15:1148–58. 15627188 10.1007/s00330-004-2565-0

[51] Debette S, Schilling S, Duperron M-G, Larsson SC, Markus HS. Clinical significance of magnetic resonance imaging markers of vascular brain injury: a systematic review and meta-analysis. JAMA Neurol. 2019;76:81–94. 30422209 10.1001/jamaneurol.2018.3122 PMC6439887

[52] Niess F, Strasser B, Hingerl L, Bader V, Frese S, Clarke WT, Duguid A, Niess E, Motyka S, Krššák M. Whole-brain deuterium metabolic imaging via concentric ring trajectory readout enables assessment of regional variations in neuronal glucose metabolism. Hum Brain Mapp. 2024;45: e26686. 38647048 10.1002/hbm.26686 PMC11034002

[53] Teichner EM, Subtirelu RC, Patil S, Al-Daoud O, Parikh C, Nguyen L, Atary J, Newberg A, Høilund-Carlsen PF, Alavi A. Bilateral carotid calcification correlates with regional cerebral glucose metabolism: insights from PET/CT imaging of patients with cardiovascular risk factors. J vasc dis. 2024;3:112–26.

[54] Nhlapho W, Atemkeng M, Brima Y, Ndogmo J-C. Bridging the gap: exploring interpretability in deep learning models for brain tumor detection and diagnosis from MRI images. Information. 2024;15:182.

[55] Deneke T, Kutyifa V, Hindricks G, Sommer P, Zeppenfeld K, Carbucicchio C, Pürerfellner H, Heinzel FR, Traykov VB, De Riva M. Pre-and post-procedural cardiac imaging (computed tomography and magnetic resonance imaging) in electrophysiology: a clinical consensus statement of the European Heart Rhythm Association and European Association of Cardiovascular Imaging of the European society of cardiology. Europace. 2024;26:108. 10.1093/europace/euae108 PMC11104536 38743765

[56] Lin K-H, Chen Y-W, Wang L-W, Wang Y-F, Hu L-H, Ting CH, Lee T-H, Lee J-C, Peng N-J. Prognostic assessment of 18F-boronophenylalanine positron emission tomography (BPA-PET) in salvage boron neutron capture therapy for malignant brain tumors. QIMS. 2024;14:4177. 38846276 10.21037/qims-23-1769 PMC11151257

[57] Balmforth C, Whittington B, Tzolos E, Bing R, Williams MC, Clark L, Corral CA, Tavares A, Dweck MR, Newby DE. Translational molecular imaging: thrombosis imaging with positron emission tomography. JNC. 2024;39: 101848. 10.1016/j.nuclcard.2024.101848 38499227

[58] Heras-Recuero E, Blázquez-Sánchez T, Landaeta-Kancev LC, Martínez de Bourio-Allona M, Torres-Roselló A, Rengifo-García F, Caraballo-Salazar C, Largo R, Castañeda S, González-Gay MÁ. Positron emission tomography/computed tomography in polymyalgia rheumatica: when and for what—a critical review. Diagnostics. 2024;14:1539. 39061676 10.3390/diagnostics14141539 PMC11275637

[59] Tong L, Cao J, Wang K, Song J, Mu J. Lanthanide-doped nanomaterials for tumor diagnosis and treatment by second near-infrared fluorescence imaging. Adv Opt Mater. 2024;12:2301767.

[60] Schmidt EL, Ou Z, Ximendes E, Cui H, Keck CH, Jaque D, Hong G. Near-infrared II fluorescence imaging. Nat Rev Methods Primers. 2024;4:23.

[61] Peper CJ, Kilgore MD, Jiang Y, Xiu Y, Xia W, Wang Y, Shi M, Zhou D, Dumont AS, Wang X. Tracing the path of disruption: 13C isotope applications in traumatic brain injury-induced metabolic dysfunction. CNS Neurosci Ther. 2024;30: e14693. 38544365 10.1111/cns.14693 PMC10973562

[62] Wu X, Yang H, Yang W, Chen X, Gao J, Gong X, Wang H, Duan Y, Wei D, Chang J. Nanoparticle-based diagnostic and therapeutic systems for brain tumors. J Mater Chem B. 2019;7:4734–50. 31389961 10.1039/c9tb00860h

[63] Schaff LR, Mellinghoff IK. Glioblastoma and other primary brain malignancies in adults: a review. JAMA. 2023;329:574–87. 36809318 10.1001/jama.2023.0023 PMC11445779

[64] Virtuoso A, D’Amico G, Scalia F, De Luca C, Papa M, Maugeri G, D’Agata V, Caruso Bavisotto C, D’Amico AG. The interplay between glioblastoma cells and tumor microenvironment: new perspectives for early diagnosis and targeted cancer therapy. Brain Sci. 2024;14:331. 38671983 10.3390/brainsci14040331 PMC11048111

[65] Indira Chandran V, Gopala S, Venkat EH, Kjolby M, Nejsum P. Extracellular vesicles in glioblastoma: a challenge and an opportunity. NPJ Precis Oncol. 2024;8:103. 38760427 10.1038/s41698-024-00600-2 PMC11101656

[66] Wu W, Klockow JL, Zhang M, Lafortune F, Chang E, Jin L, Wu Y, Daldrup-Link HE. Glioblastoma multiforme (GBM): an overview of current therapies and mechanisms of resistance. Pharmacol Res. 2021;171: 105780. 34302977 10.1016/j.phrs.2021.105780 PMC8384724

[67] Ostrom QT, Patil N, Cioffi G, Waite K, Kruchko C, Barnholtz-Sloan JS. CBTRUS statistical report: primary brain and other central nervous system tumors diagnosed in the United States in 2013–2017. Neuro Oncol. 2020;22:iv1–96. 33123732 10.1093/neuonc/noaa200 PMC7596247

[68] Caccese M, Padovan M, D’Avella D, Chioffi F, Gardiman MP, Berti F, Busato F, Bellu L, Bergo E, Zoccarato M, et al. Anaplastic astrocytoma: state of the art and future directions. Crit Rev Oncol Hematol. 2020;153: 103062. 32717623 10.1016/j.critrevonc.2020.103062

[69] Achrol AS, Rennert RC, Anders C, Soffietti R, Ahluwalia MS, Nayak L, Peters S, Arvold ND, Harsh GR, Steeg PS, Chang SD. Brain metastases. Nat Rev Dis Primers. 2019;5:5. 30655533 10.1038/s41572-018-0055-y

[70] Aldoghachi AF, Aldoghachi AF, Breyne K, Ling K-H, Cheah P-S. Recent advances in the therapeutic strategies of glioblastoma multiforme. Neurosci. 2022;491:240–70. 10.1016/j.neuroscience.2022.03.030 35395355

[71] Im JH, Hong JB, Kim SH, Choi J, Chang JH, Cho J, Suh C-O. Recurrence patterns after maximal surgical resection and postoperative radiotherapy in anaplastic gliomas according to the new 2016 WHO classification. Sci Rep. 2018;8:777. 29335518 10.1038/s41598-017-19014-1 PMC5768800

[72] Anand U, Dey A, Chandel AKS, Sanyal R, Mishra A, Pandey DK, De Falco V, Upadhyay A, Kandimalla R, Chaudhary A, et al. Cancer chemotherapy and beyond: current status, drug candidates, associated risks and progress in targeted therapeutics. Genes Dis. 2023;10:1367–401. 37397557 10.1016/j.gendis.2022.02.007 PMC10310991

[73] Cheng Y, Morshed RA, Auffinger B, Tobias AL, Lesniak MS. Multifunctional nanoparticles for brain tumor imaging and therapy. Adv Drug Deliv Rev. 2014;66:42–57. 24060923 10.1016/j.addr.2013.09.006 PMC3948347

[74] Warszyńska M, Repetowski P, Dąbrowski JM. Photodynamic therapy combined with immunotherapy: recent advances and future research directions. Coord Chem Rev. 2023;495: 215350.

[75] Belete TM. The current status of gene therapy for the treatment of cancer. Biologics. 2021;15:67–77. 33776419 10.2147/BTT.S302095 PMC7987258

[76] Kaifi R. A review of recent advances in brain tumor diagnosis based on AI-based classification. Diagnostics (Basel). 2023;13:3007. 37761373 10.3390/diagnostics13183007 PMC10527911

[77] Bai J-W, Qiu S-Q, Zhang G-J. Molecular and functional imaging in cancer-targeted therapy: current applications and future directions. Signal Transduct Target Ther. 2023;8:89. 36849435 10.1038/s41392-023-01366-y PMC9971190

[78] Wang K, Du Y, Zhang Z, He K, Cheng Z, Yin L, Dong D, Li C, Li W, Hu Z, et al. Fluorescence image-guided tumour surgery. Nat Rev Bioeng. 2023;1:161–79.

[79] Jo S, Sun I-C, Ahn C-H, Lee S, Kim K. Recent trend of ultrasound-mediated nanoparticle delivery for brain imaging and treatment. ACS Appl Mater Interfaces. 2023;15:120–37. 35184560 10.1021/acsami.1c22803

[80] Knox EG, Aburto MR, Clarke G, Cryan JF, O’Driscoll CM. The blood-brain barrier in aging and neurodegeneration. Mol Psychiatry. 2022;27:2659–73. 35361905 10.1038/s41380-022-01511-z PMC9156404

[81] Beygi M, Oroojalian F, Azizi-Arani S, Hosseini SS, Mokhtarzadeh A, Kesharwani P, Sahebkar A. Multifunctional nanotheranostics for overcoming the blood-brain barrier. Adv Funct Mater. 2024;34:2310881.

[82] Robey RW, Pluchino KM, Hall MD, Fojo AT, Bates SE, Gottesman MM. Revisiting the role of ABC transporters in multidrug-resistant cancer. Nat Rev Cancer. 2018;18:452–64. 29643473 10.1038/s41568-018-0005-8 PMC6622180

[83] Evans PG, Sokolska M, Alves A, Harrison IF, Ohene Y, Nahavandi P, Ismail O, Miranda E, Lythgoe MF, Thomas DL, Wells JA. Non-invasive MRI of blood-cerebrospinal fluid barrier function. Nat Commun. 2020;11:2081. 32350278 10.1038/s41467-020-16002-4 PMC7190825

[84] Song Y, Hu C, Fu Y, Gao H. Modulating the blood–brain tumor barrier for improving drug delivery efficiency and efficacy. VIEW. 2022;3:20200129.

[85] Schaaf MB, Garg AD, Agostinis P. Defining the role of the tumor vasculature in antitumor immunity and immunotherapy. Cell Death Dis. 2018;9:115. 29371595 10.1038/s41419-017-0061-0 PMC5833710

[86] Upadhyay RK. Drug delivery systems, CNS protection, and the blood brain barrier. Biomed Res Int. 2014;2014: 869269. 25136634 10.1155/2014/869269 PMC4127280

[87] Moore C, Jokerst JV. Strategies for image-guided therapy, surgery, and drug delivery using photoacoustic imaging. Theranostics. 2019;9:1550–71. 31037123 10.7150/thno.32362 PMC6485201

[88] Liu Z, Li J, Chen W, Liu L, Yu F. Light and sound to trigger the Pandora’s box against breast cancer: a combination strategy of sonodynamic, photodynamic and photothermal therapies. Biomater. 2020;232: 119685. 10.1016/j.biomaterials.2019.119685 31918219

[89] Park B, Park S, Kim J, Kim C. Listening to drug delivery and responses via photoacoustic imaging. Adv Drug Deliv Rev. 2022;184: 114235. 35346776 10.1016/j.addr.2022.114235

[90] Guo B, Sheng Z, Hu D, Liu C, Zheng H, Liu B. Through scalp and Skull NIR-II photothermal therapy of deep orthotopic brain tumors with precise photoacoustic imaging guidance. Adv Mater. 2018;30:1802591. 10.1002/adma.201802591 30129690

[91] Wang D, Wu Y, Xia J. Review on photoacoustic imaging of the brain using nanoprobes. Neurophotonics. 2016;3: 010901. 26740961 10.1117/1.NPh.3.1.010901 PMC4699324

[92] Okumura K, Yoshida K, Yoshioka K, Aki S, Yoneda N, Inoue D, Kitao A, Ogi T, Kozaka K, Minami T, et al. Photoacoustic imaging of tumour vascular permeability with indocyanine green in a mouse model. Eur Radiol Exp. 2018;2:5. 29708213 10.1186/s41747-018-0036-7 PMC5909364

[93] Abbas M. Potential role of nanoparticles in treating the accumulation of amyloid-beta peptide in Alzheimer’s patients. Polymers (Basel). 2021;13:1051. 33801619 10.3390/polym13071051 PMC8036916

[94] Wang S, Sheng Z, Yang Z, Hu D, Long X, Feng G, Liu Y, Yuan Z, Zhang J, Zheng H, Zhang X. Activatable small-molecule photoacoustic probes that cross the blood-brain barrier for visualization of copper(II) in mice with Alzheimer’s disease. Angew Chem Int Edit. 2019;58:12415–9. 10.1002/anie.201904047 31309679

[95] Liu L, Chen Q, Wen L, Li C, Qin H, Xing D. Photoacoustic therapy for precise eradication of glioblastoma with a tumor site blood-brain barrier permeability upregulating nanoparticle. Adv Funct Mater. 2019;29:1808601.

[96] Al-Thani AN, Jan AG, Abbas M, Geetha M, Sadasivuni KK. Nanoparticles in cancer theragnostic and drug delivery: a comprehensive review. Life Sci. 2024;352: 122899. 38992574 10.1016/j.lfs.2024.122899

[97] Zhong J, Yang S, Wen L, Xing D. Imaging-guided photoacoustic drug release and synergistic chemo-photoacoustic therapy with paclitaxel-containing nanoparticles. JCR. 2016;226:77–87. 10.1016/j.jconrel.2016.02.010 26860283

[98] Geng X, Gao D, Hu D, Liu Q, Liu C, Yuan Z, Zhang X, Liu X, Sheng Z, Wang X, Zheng H. Active-targeting NIR-II phototheranostics in multiple tumor models using platelet-camouflaged nanoprobes. ACS Appl Mater Interfaces. 2020;12:55624–37. 33269904 10.1021/acsami.0c16872

[99] Farajollahi A, Baharvand M. Advancements in photoacoustic imaging for cancer diagnosis and treatment. Int J Pharm. 2024;665: 124736. 39326479 10.1016/j.ijpharm.2024.124736

[100] Jeevarathinam AS, Pai N, Huang K, Hariri A, Wang J, Bai Y, Wang L, Hancock T, Keys S, Penny W, Jokerst JV. A cellulose-based photoacoustic sensor to measure heparin concentration and activity in human blood samples. Biosens Bioelectron. 2019;126:831–7. 30602265 10.1016/j.bios.2018.11.052 PMC6357780

[101] Oraevsky AA, Jacques SL, Tittel FK. Determination of tissue optical properties by piezoelectric detection of laser-induced stress waves. Laser-Tissue Interact. 1993;1882:86–101.

[102] Oraevsky AA, Jacques SL, Tittel FK. Measurement of tissue optical properties by time-resolved detection of laser-induced transient stress. Appl Opt. 1997;36:402–15. 18250688 10.1364/ao.36.000402

[103] Wada K, Masujima T, Yoshida H, Murakami T, Yata N, Imai H. Application of photoacoustic microscopy to analysis of biological components in tissue sections. Chem Pharm Bull. 1986;34:1688–93. 10.1248/cpb.34.1688 3719869

[104] Maslov K, Stoica G, Wang LV. In vivo dark-field reflection-mode photoacoustic microscopy. Opt Lett. 2005;30:625–7. 15791997 10.1364/ol.30.000625

[105] Manohar S, Razansky D. Photoacoustics: a historical review. Adv Opt Photonics. 2016;8:586–617.

[106] Yao J, Wang LV. Sensitivity of photoacoustic microscopy. Photoacoustics. 2014;2:87–101. 25302158 10.1016/j.pacs.2014.04.002 PMC4182819

[107] Razansky D, Buehler A, Ntziachristos V. Volumetric real-time multispectral optoacoustic tomography of biomarkers. Nat Protoc. 2011;6:1121–9. 21738125 10.1038/nprot.2011.351

[108] Deán-Ben X, Gottschalk S, Mc Larney B, Shoham S, Razansky D. Advanced optoacoustic methods for multiscale imaging of in vivo dynamics. Chem Soc Rev. 2017;46:2158–98. 28276544 10.1039/c6cs00765a PMC5460636

[109] Zhang F, Zhang J, Shen Y, Gao Z, Yang C, Liang M, Gao F, Liu L, Zhao H, Gao F. Photoacoustic digital brain and deep-learning-assisted image reconstruction. Photoacoustics. 2023;31: 100517. 37292518 10.1016/j.pacs.2023.100517 PMC10244697

[110] Bodea S-V, Westmeyer GG. Photoacoustic neuroimaging—perspectives on a maturing imaging technique and its applications in neuroscience. Front Neurosci. 2021;15:655247. 34220420 10.3389/fnins.2021.655247 PMC8253050

[111] Maslov K, Zhang HF, Hu S, Wang LV. Optical-resolution photoacoustic microscopy for in vivo imaging of single capillaries. Opt Lett. 2008;33:929–31. 18451942 10.1364/ol.33.000929

[112] Hu S, Maslov K, Wang LV. Second-generation optical-resolution photoacoustic microscopy with improved sensitivity and speed. Opt Lett. 2011;36:1134–6. 21479007 10.1364/OL.36.001134 PMC3076123

[113] Hu S, Wang LV. Optical-resolution photoacoustic microscopy: auscultation of biological systems at the cellular level. Biophys J. 2013;105:841–7. 23972836 10.1016/j.bpj.2013.07.017 PMC3752103

[114] Yao J, Wang LV. Multi-scale multi-contrast photoacoustic microscopy. Front Opt. 2013. 10.1364/FIO.2013.FM4A.1.

[115] Seeger M, Soliman D, Aguirre J, Diot G, Wierzbowski J, Ntziachristos V. Pushing the boundaries of optoacoustic microscopy by total impulse response characterization. Nat Commun. 2020;11:2910. 32518250 10.1038/s41467-020-16565-2 PMC7283257

[116] Bell KL, Hajireza P, Shi W, Zemp RJ. Temporal evolution of low-coherence reflectrometry signals in photoacoustic remote sensing microscopy. Appl Opt. 2017;56:5172–81. 29047569 10.1364/AO.56.005172

[117] Hajireza P, Shi W, Bell K, Paproski RJ, Zemp RJ. Non-interferometric photoacoustic remote sensing microscopy. Light sci appl. 2017;6:e16278–e16278. 30167263 10.1038/lsa.2016.278 PMC6062239

[118] Li M-L, Zhang HF, Maslov K, Stoica G, Wang LV. Improved in vivo photoacoustic microscopy based on a virtual-detector concept. Opt Lett. 2006;31:474–6. 16496891 10.1364/ol.31.000474

[119] Zhang HF, Maslov K, Stoica G, Wang LV. Functional photoacoustic microscopy for high-resolution and noninvasive in vivo imaging. Nat Biotechnol. 2006;24:848–51. 16823374 10.1038/nbt1220

[120] Park S, Lee C, Kim J, Kim C. Acoustic resolution photoacoustic microscopy. Biomed Eng Lett. 2014;4:213–22.

[121] Omar M, Soliman D, Gateau J, Ntziachristos V. Ultrawideband reflection-mode optoacoustic mesoscopy. Opt Lett. 2014;39:3911–4. 24978769 10.1364/OL.39.003911

[122] Soliman D, Tserevelakis GJ, Omar M, Ntziachristos V. Combining microscopy with mesoscopy using optical and optoacoustic label-free modes. Sci Rep. 2015;5:12902. 26306396 10.1038/srep12902 PMC4549672

[123] Kruger RA, Liu P, Fang YR, Appledorn CR. Photoacoustic ultrasound (PAUS)—reconstruction tomography. Med Phys. 1995;22:1605–9. 8551984 10.1118/1.597429

[124] Hoelen C, De Mul F, Pongers R, Dekker A. Three-dimensional photoacoustic imaging of blood vessels in tissue. Opt Lett. 1998;23:648–50. 18084605 10.1364/ol.23.000648

[125] Buehler A, Herzog E, Razansky D, Ntziachristos V. Video rate optoacoustic tomography of mouse kidney perfusion. Opt Lett. 2010;35:2475–7. 20634868 10.1364/OL.35.002475

[126] Lutzweiler C, Razansky D. Optoacoustic imaging and tomography: reconstruction approaches and outstanding challenges in image performance and quantification. Sensors. 2013;13:7345–84. 23736854 10.3390/s130607345 PMC3715274

[127] Sun A, Guo H, Gan Q, Yang L, Liu Q, Xi L. Evaluation of visible NIR-I and NIR-II light penetration for photoacoustic imaging in rat organs. Opt Express. 2020;28:9002–13. 32225514 10.1364/OE.389714

[128] Wilson K, Homan K, Emelianov S. Biomedical photoacoustics beyond thermal expansion using triggered nanodroplet vaporization for contrast-enhanced imaging. Nat Commun. 2012;3:618. 22233628 10.1038/ncomms1627

[129] Li L, Wang LV. Recent advances in photoacoustic tomography. BME Front. 2021;2021:9823268. 37041754 10.34133/2021/9823268 PMC10085577

[130] Feng G, Zhang G-Q, Ding D. Design of superior phototheranostic agents guided by Jablonski diagrams. Chem Soc Rev. 2020;49:8179–234. 33196726 10.1039/d0cs00671h

[131] Yang C, Lan H, Gao F. Accelerated photoacoustic tomography reconstruction via recurrent inference machines. In: Yang C, editor. 2019 41st Annual international conference of the IEEE engineering in medicine and biology society (EMBC). Berlin: IEEE; 2019. 10.1109/EMBC.2019.8856290 31947300

[132] Yang C, Lan H, Gao F, Gao F. Review of deep learning for photoacoustic imaging. Photoacoustics. 2021;21: 100215. 33425679 10.1016/j.pacs.2020.100215 PMC7779783

[133] Weber J, Beard PC, Bohndiek SE. Contrast agents for molecular photoacoustic imaging. Nat Methods. 2016;13:639–50. 27467727 10.1038/nmeth.3929

[134] Piper K, Kumar JI, Domino J, Tuchek C, Vogelbaum MA. Consensus review on strategies to improve delivery across the BBB including focused ultrasound. Neuro Oncol. 2024. 10.1093/neuonc/noae087. 38770775 10.1093/neuonc/noae087 PMC11376463

[135] Razansky D, Klohs J, Ni R. Multi-scale optoacoustic molecular imaging of brain diseases. Eur J Nucl Med Mol Imaging. 2021;48:4152–70. 33594473 10.1007/s00259-021-05207-4 PMC8566397

[136] Kasten BB, Jiang K, Cole D, Jani A, Udayakumar N, Gillespie GY, Lu G, Dai T, Rosenthal EL, Markert JM. Targeting MMP-14 for dual PET and fluorescence imaging of glioma in preclinical models. Eur J Nucl Med Mol Imaging. 2020;47:1412–26. 31773232 10.1007/s00259-019-04607-x PMC7968515

[137] Roberts S, Seeger M, Jiang Y, Mishra A, Sigmund F, Stelzl A, Lauri A, Symvoulidis P, Rolbieski H, Preller M. Calcium sensor for photoacoustic imaging. JACS. 2018;140:2718–21. 10.1021/jacs.7b03064 28945084

[138] Qian Y, Piatkevich KD, Mc Larney B, Abdelfattah AS, Mehta S, Murdock MH, Gottschalk S, Molina RS, Zhang W, Chen Y. A genetically encoded near-infrared fluorescent calcium ion indicator. Nat Methods. 2019;16:171–4. 30664778 10.1038/s41592-018-0294-6 PMC6393164

[139] Mishra K, Stankevych M, Fuenzalida-Werner JP, Grassmann S, Gujrati V, Huang Y, Klemm U, Buchholz VR, Ntziachristos V, Stiel AC. Multiplexed whole-animal imaging with reversibly switchable optoacoustic proteins. Sci Adv. 2020;6:6293. 10.1126/sciadv.aaz6293 PMC7292636 32582850

[140] Farhadi A, Sigmund F, Westmeyer GG, Shapiro MG. Genetically encodable materials for non-invasive biological imaging. Nat Mater. 2021;20:585–92. 33526879 10.1038/s41563-020-00883-3 PMC8606175

[141] Qu X, Hu Q, Song Z, Sun Z, Zhang B, Zhong J, Cao X, Liu Y, Zhao B, Liu Z. Adsorption and desorption mechanisms on graphene oxide nanosheets: Kinetics and tuning. Innov. 2021;2:100137. 10.1016/j.xinn.2021.100137 PMC8454550 34557777

[142] Shemetov AA, Monakhov MV, Zhang Q, Canton-Josh JE, Kumar M, Chen M, Matlashov ME, Li X, Yang W, Nie L. A near-infrared genetically encoded calcium indicator for in vivo imaging. Nat Biotechnol. 2021;39:368–77. 33106681 10.1038/s41587-020-0710-1 PMC7956128

[143] Zhan C, Huang Y, Lin G, Huang S, Zeng F, Wu S. A Gold Nanocage/cluster hybrid structure for whole-body multispectral optoacoustic tomography imaging, EGFR inhibitor delivery, and photothermal therapy. Small. 2019;15:1900309. 10.1002/smll.201900309 31245925

[144] Xu Y, Zhang Y, Li J, An J, Li C, Bai S, Sharma A, Deng G, Kim JS, Sun Y. NIR-II emissive multifunctional AIEgen with single laser-activated synergistic photodynamic/photothermal therapy of cancers and pathogens. Biomater. 2020;259: 120315. 10.1016/j.biomaterials.2020.120315 32836057

[145] Cheng H, Wang X, Liu X, Wang X, Wen H, Cheng Y, Xie A, Shen Y, Tang R, Zhu M. An effective NIR laser/tumor-microenvironment co-responsive cancer theranostic nanoplatform with multi-modal imaging and therapies. Nanoscale. 2021;13:10816–28. 34113940 10.1039/d1nr01645h

[146] Fan Z, Liu H, Xue Y, Lin J, Fu Y, Xia Z, Pan D, Zhang J, Qiao K, Zhang Z. Reversing cold tumors to hot: an immunoadjuvant-functionalized metal-organic framework for multimodal imaging-guided synergistic photo-immunotherapy. Bioact Mater. 2021;6:312–25. 32954050 10.1016/j.bioactmat.2020.08.005 PMC7475520

[147] Joseph J, Baumann KN, Postigo A, Bollepalli L, Bohndiek SE, Hernández-Ainsa S. DNA-based nanocarriers to enhance the optoacoustic contrast of tumors in vivo. Adv Healthc Mater. 2021;10:2001739. 10.1002/adhm.202001739 33191661

[148] Qi S, Zhang Y, Liu G, Chen J, Li X, Zhu Q, Yang Y, Wang F, Shi J, Lee C-S. Plasmonic-doped melanin-mimic for CXCR4-targeted NIR-II photoacoustic computed tomography-guided photothermal ablation of orthotopic hepatocellular carcinoma. Acta Biomater. 2021;129:245–57. 34082093 10.1016/j.actbio.2021.05.034

[149] Ye Z, Bao Y, Chen Z, Ye H, Feng Z, Li Y, Zeng Y, Pan Z, Ouyang D, Zhang K, et al. Recent advances in the metal/organic hybrid nanomaterials for cancer theranostics. Coord Chem Rev. 2024;504: 215654.

[150] Lim SH, Yee GT, Khang D. Nanoparticle-based combinational strategies for overcoming the blood-brain barrier and blood-tumor barrier. Int J Nanomedicine. 2024;19:2529–52. 38505170 10.2147/IJN.S450853 PMC10949308

[151] Li Y, Li L, Zhu L, Maslov K, Shi J, Hu P, Bo E, Yao J, Liang J, Wang L. Snapshot photoacoustic topography through an ergodic relay for high-throughput imaging of optical absorption. Nat Photonics. 2020;14:164–70. 34178097 10.1038/s41566-019-0576-2 PMC8223468

[152] Attia ABE, Ho CJH, Chandrasekharan P, Balasundaram G, Tay HC, Burton NC, Chuang KH, Ntziachristos V, Olivo M. Multispectral optoacoustic and MRI coregistration for molecular imaging of orthotopic model of human glioblastoma. J Biophotonics. 2016;9:701–8. 27091626 10.1002/jbio.201500321

[153] Sun Y, Wang Y, Li W, Li C. Real-time dual-modal photoacoustic and fluorescence small animal imaging. Photoacoustics. 2024;36: 100593. 38352643 10.1016/j.pacs.2024.100593 PMC10862394

[154] Yang Q, Ye W, Luo D, Xing J, Xiao Q, Wu H, Yao Y, Wang G, Yang L, Guo D, et al. Neuroprotective effects of anti-TRAIL-ICG nanoagent and its multimodal imaging evaluation in cerebral ischemia-reperfusion injury. Mater Today Bio. 2024;26: 101094. 38854952 10.1016/j.mtbio.2024.101094 PMC11157279

[155] Leng F, Edison P. Neuroinflammation and microglial activation in Alzheimer disease: where do we go from here? Nat Rev Neurol. 2021;17:157–72. 33318676 10.1038/s41582-020-00435-y

[156] McAlpine CS, Park J, Griciuc A, Kim E, Choi SH, Iwamoto Y, Kiss MG, Christie KA, Vinegoni C, Poller WC. Astrocytic interleukin-3 programs microglia and limits Alzheimer’s disease. Nature. 2021;595:701–6. 34262178 10.1038/s41586-021-03734-6 PMC8934148

[157] Cabral-Pacheco GA, Garza-Veloz I, Castruita-De la Rosa C, Ramirez-Acuña JM, Perez-Romero BA, Guerrero-Rodriguez JF, Martinez-Avila N, Martinez-Fierro ML. The roles of matrix metalloproteinases and their inhibitors in human diseases. Int J Mol Sci. 2020;21:9739. 33419373 10.3390/ijms21249739 PMC7767220

[158] Ni R, Vaas M, Ren W, Klohs J. Noninvasive detection of acute cerebral hypoxia and subsequent matrix-metalloproteinase activity in a mouse model of cerebral ischemia using multispectral-optoacoustic-tomography. Neurophotonics. 2018;5:015005–015005. 29531962 10.1117/1.NPh.5.1.015005 PMC5829216

[159] Ni R, Dean-Ben XL, Kirschenbaum D, Rudin M, Chen Z, Crimi A, Voigt FF, Nilsson KPR, Helmchen F, Nitsch R. Whole brain optoacoustic tomography reveals strain-specific regional beta-amyloid densities in Alzheimer’s disease amyloidosis models. bioRxiv. 2020;72:287.

[160] Ni R, Chen Z, Deán-Ben XL, Voigt FF, Kirschenbaum D, Shi G, Villois A, Zhou Q, Crimi A, Arosio P, et al. Multiscale optical and optoacoustic imaging of amyloid-β deposits in mice. Nat Biomed Eng. 2022;6:1031–44. 35835994 10.1038/s41551-022-00906-1

[161] Vagenknecht P, Luzgin A, Ono M, Ji B, Higuchi M, Noain D, Maschio CA, Sobek J, Chen Z, Konietzko U. Non-invasive imaging of tau-targeted probe uptake by whole brain multi-spectral optoacoustic tomography. Eur J Nucl Med Mol Imaging. 2022;49:2137–52. 35128565 10.1007/s00259-022-05708-w PMC9165274

[162] Mathivanan SK, Sonaimuthu S, Murugesan S, Rajadurai H, Shivahare BD, Shah MA. Employing deep learning and transfer learning for accurate brain tumor detection. Sci Rep. 2024;14:7232. 38538708 10.1038/s41598-024-57970-7 PMC10973383

[163] Davis KM, Ryan JL, Aaron VD, Sims JB. PET and SPECT imaging of the brain: history, technical considerations, applications, and radiotracers. Semin Ultrasound CT MR. 2020;41:521–9. 33308491 10.1053/j.sult.2020.08.006

[164] Sharma SD. Radiation environment in medical facilities. In: Aswal DK, editor. Handbook on radiation environment, volume 2: dose measurements. Singapore: Springer Nature; 2024.

[165] Wang J, Wang Y, Zhong L, Yan F, Zheng H. Nanoscale contrast agents: a promising tool for ultrasound imaging and therapy. Adv Drug Deliv Rev. 2024;207: 115200. 38364906 10.1016/j.addr.2024.115200

[166] Geng Y, Zou H, Li Z, Wu H. Recent advances in nanomaterial-driven strategies for diagnosis and therapy of vascular anomalies. J Nanobiotechnology. 2024;22:120. 38500178 10.1186/s12951-024-02370-2 PMC10949774

[167] Lang Y, Jiang Z, Sun L, Xiang L, Ren L. Hybrid-supervised deep learning for domain transfer 3D protoacoustic image reconstruction. Phys Med Biol. 2024;69: 085007. 10.1088/1361-6560/ad3327 PMC11076107 38471184

[168] Huang Z, Tian H, Luo H, Yang K, Chen J, Li G, Ding Z, Luo Y, Tang S, Xu J. Assessment of oxygen saturation in breast lesions using photoacoustic imaging: correlation with benign and malignant disease. Clin Breast Cancer. 2024. 10.1016/j.clbc.2024.01.006. 38423948 10.1016/j.clbc.2024.01.006

[169] Kang H, Lee SW, Park SM, Cho SW, Lee JY, Kim CS, Lee TG. Real-time functional optical-resolution photoacoustic microscopy using high-speed alternating illumination at 532 and 1064 nm. J Biophotonics. 2018;11: e201700210. 10.1002/jbio.201700210 28945324

[170] Jathoul AP, Laufer J, Ogunlade O, Treeby B, Cox B, Zhang E, Johnson P, Pizzey AR, Philip B, Marafioti T, et al. Deep in vivo photoacoustic imaging of mammalian tissues using a tyrosinase-based genetic reporter. Nat Photonics. 2015;9:239–46.

[171] Zhou Y, Wang D, Zhang Y, Chitgupi U, Geng J, Wang Y, Zhang Y, Cook TR, Xia J, Lovell JF. A phosphorus phthalocyanine formulation with intense absorbance at 1000 nm for deep optical imaging. Theranostics. 2016;6:688–97. 27022416 10.7150/thno.14555 PMC4805663

[172] Liu X, Duan Y, Liu B. Nanoparticles as contrast agents for photoacoustic brain imaging. Aggregate. 2021;2:4–19.

[173] Li LP, Ren XF, Bai PR, Liu Y, Xu WY, Xie J. Near-infrared emission carbon dots for bio-imaging applications. New Carbon Mater. 2021;36:632–8.

[174] Liu Y, Liu J, Zhang J, Li X, Lin F, Zhou N, Yang B, Lu L. Noninvasive brain tumor imaging using red emissive carbonized polymer dots across the blood-brain barrier. ACS Omega. 2018;3:7888–96. 30087926 10.1021/acsomega.8b01169 PMC6072250

[175] Neelamraju PM, Gundepudi K, Sanki PK, Busi KB, Mistri TK, Sangaraju S, Dalapati GK, Ghosh KK, Ghosh S, Ball WB, Chakrabortty S. Potential applications for photoacoustic imaging using functional nanoparticles: a comprehensive overview. Heliyon. 2024;10: e34654. 39166037 10.1016/j.heliyon.2024.e34654 PMC11334826

[176] Duan Y, Hu D, Guo B, Shi Q, Wu M, Xu S, Kenry LX, Jiang J, Sheng Z. Nanostructural control enables optimized photoacoustic–fluorescence–magnetic resonance multimodal imaging and photothermal therapy of brain tumor. Adv Funct Mater. 2020;30:1907077.

[177] Chi S, Wang C, Liu Z. Biomimetic nanocomposites for glioma imaging and therapy. Chem Eur J. 2024. 10.1002/chem.202304338. 38538540 10.1002/chem.202304338

[178] Gupta M, Singh SP. Nanoparticles for multimodal imaging and theranostic applications in cancer diagnosis and treatment. J pharmacogn phytochem. 2024;13:236–43.

[179] Ullah Z, Roy S, Gu J, Ko Soe S, Jin J, Guo B. NIR-II fluorescent probes for fluorescence-imaging-guided tumor surgery. Biosensors. 2024;14:282. 38920586 10.3390/bios14060282 PMC11201439

[180] Zhang Y, Miao S, Li Q, Zhou T, Hu J, Deng Y, Li Z, Cao Z, Huang X, Sheng Z. Semiconducting polymers based on asymmetric thiadiazoloquinoxaline for augmented in vivo NIR-II photoacoustic imaging. Biomacromol. 2024. 10.1021/acs.biomac.4c00258. 10.1021/acs.biomac.4c00258 38693895

[181] Hu J, Zhou T, Li Q, Zhou F, Miao S, Li Z, Zhang Y, Deng Y, Cao Y, Xiao X. Semiconducting polymers containing a highly absorptive, bay-shaped building block for effective NIR photoacoustic imaging. ACS Appl Polym Mater. 2024;6:4687–95.

[182] Janib SM, Moses AS, MacKay JA. Imaging and drug delivery using theranostic nanoparticles. Adv Drug Deliv Rev. 2010;62:1052–63. 20709124 10.1016/j.addr.2010.08.004 PMC3769170

[183] Zha S, Liu H, Li H, Li H, Wong K-L, All AH. Functionalized nanomaterials capable of crossing the blood-brain barrier. ACS Nano. 2024;18:1820–45. 38193927 10.1021/acsnano.3c10674 PMC10811692

[184] Smilowitz HM, Meyers A, Rahman K, Dyment NA, Sasso D, Xue C, Oliver DL, Lichtler A, Deng X, Ridwan SM, et al. Intravenously-injected gold nanoparticles (AuNPs) access intracerebral F98 rat gliomas better than AuNPs infused directly into the tumor site by convection enhanced delivery. Int J Nanomedicine. 2018;13:3937–48. 30013346 10.2147/IJN.S154555 PMC6038872

[185] Sela H, Cohen H, Elia P, Zach R, Karpas Z, Zeiri Y. Spontaneous penetration of gold nanoparticles through the blood brain barrier (BBB). J Nanobiotechnology. 2015;13:1–9. 26489846 10.1186/s12951-015-0133-1 PMC4618365

[186] Hu H, Yuan M, Chen J, Fan T, Nguyen N, Madison CA, Yan T, Xiao Z, Li Y, Eitan S. Pharmacokinetic modeling of solid and hollow gold-coated superparamagnetic iron oxide nanoparticles for brain-targeted therapeutics: prediction and experiment. Adv Compos Hybrid Mater. 2024;7:76.

[187] Thawani JP, Amirshaghaghi A, Yan L, Stein JM, Liu J, Tsourkas A. Photoacoustic-guided surgery with indocyanine green-coated superparamagnetic Iron oxide nanoparticle clusters. Small. 2017;13:1701300. 10.1002/smll.201701300 PMC5884067 28748623

[188] Liu C, Chen J, Zhu Y, Gong X, Zheng R, Chen N, Chen D, Yan H, Zhang P, Zheng H, et al. Highly sensitive MoS2–indocyanine green hybrid for photoacoustic imaging of orthotopic brain glioma at deep site. Nano-Micro Lett. 2018;10:48. 10.1007/s40820-018-0202-8 PMC6199097 30393697

[189] Wu M, Chen W, Chen Y, Zhang H, Liu C, Deng Z, Sheng Z, Chen J, Liu X, Yan F, Zheng H. Focused ultrasound-augmented delivery of biodegradable multifunctional nanoplatforms for imaging-guided brain tumor treatment. Adv Sci. 2018;5:1700474. 10.1002/advs.201700474 PMC5908350 29721406

[190] Chen J, Liu C, Hu D, Wang F, Wu H, Gong X, Liu X, Song L, Sheng Z, Zheng H. Single-layer MoS2 nanosheets with amplified photoacoustic effect for highly sensitive photoacoustic imaging of orthotopic brain tumors. Adv Funct Mater. 2016;26:8715–25.

[191] Liu T, Wang C, Gu X, Gong H, Cheng L, Shi X, Feng L, Sun B, Liu Z. Drug delivery with PEGylated MoS2 nano-sheets for combined photothermal and chemotherapy of cancer. Adv Mater (Deerfield Beach, Fla). 2014;26:3433–40. 10.1002/adma.201305256 24677423

[192] Xu Y, Wang S, Chen Z, Hu R, Li S, Zhao Y, Liu L, Qu J. Highly stable organic photothermal agent based on near-infrared-II fluorophores for tumor treatment. J Nanobiotechnology. 2021;19:1–14. 33541369 10.1186/s12951-021-00782-y PMC7863535

[193] Wen G, Li X, Zhang Y, Han X, Xu X, Liu C, Chan KW, Lee C-S, Yin C, Bian L. Effective phototheranostics of brain tumor assisted by near-infrared-II light-responsive semiconducting polymer nanoparticles. ACS Appl Mater Interfaces. 2020;12:33492–9. 32627525 10.1021/acsami.0c08562

[194] Fan Q, Cheng K, Yang Z, Zhang R, Yang M, Hu X, Ma X, Bu L, Lu X, Xiong X, et al. Photoacoustic imaging: perylene-diimide-based nanoparticles as highly efficient photoacoustic agents for deep brain tumor imaging in living mice. Adv Mater. 2015;27:774–774. 10.1002/adma.201402972 PMC4347809 25376906

[195] Yin C, Wen G, Liu C, Yang B, Lin S, Huang J, Zhao P, Hong S, Wong D, Zhang K, Chen X, Li G, Jiang X, Huang J, Pu K, Wang L, Bian L. Organic semiconducting polymer nanoparticles for photoacoustic labeling and tracking of stem cells in the Second Near-Infrared Window. ACS Nano 2018;12(12):12201–11. 10.1021/acsnano.8b05906. 10.1021/acsnano.8b05906 30433761

[196] Chen Z, Gezginer I, Zhou Q, Tang L, Deán-Ben XL, Razansky D. Multimodal optoacoustic imaging: methods and contrast materials. Chem Soc Rev. 2024. 10.1039/D3CS00565H. 38738633 10.1039/d3cs00565h PMC11181994

[197] Karakatsani ME, Beltrán HE, Chen Z, Shoham S, Deán-Ben XL, Razansky D. Shedding light on ultrasound in action: optical and optoacoustic monitoring of ultrasound brain interventions. Adv Drug Deliv Rev. 2024;205:115177. 38184194 10.1016/j.addr.2023.115177 PMC11298795

[198] Zhao T, Desjardins AE, Ourselin S, Vercauteren T, Xia W. Minimally invasive photoacoustic imaging: current status and future perspectives. Photoacoustics. 2019;16: 100146. 31871889 10.1016/j.pacs.2019.100146 PMC6909166

[199] Kim J, Lee J, Choi S, Lee H, Yang J, Jeon H, Sung M, Kim WJ, Kim C. 3d multiparametric photoacoustic computed tomography of primary and metastatic tumors in living mice. ACS Nano. 2024. 10.1021/acsnano.3c12551. 38941553 10.1021/acsnano.3c12551 PMC11256897

[200] Yang S, Hu S. Perspectives on endoscopic functional photoacoustic microscopy. Appl Phys Lett. 2024;125:030502. 39022117 10.1063/5.0201691 PMC11251735

[201] Kim M, Lee J-H, Nam J-M. Plasmonic photothermal nanoparticles for biomedical applications. Adv Sci. 2019;6:1900471. 10.1002/advs.201900471 PMC6724476 31508273

[202] Krauze AV, Myrehaug SD, Chang MG, Holdford DJ, Smith S, Shih J, Tofilon PJ, Fine HA, Camphausen K. A phase 2 study of concurrent radiation therapy, temozolomide, and the histone deacetylase inhibitor valproic acid for patients with glioblastoma. Int J Radiat Oncol Biol Phys. 2015;92:986–92. 26194676 10.1016/j.ijrobp.2015.04.038 PMC4510472

[203] Ahamed J, Jaswanth Gowda BH, Almalki WH, Gupta N, Sahebkar A, Kesharwani P. Recent advances in nanoparticle-based approaches for the treatment of brain tumors: opportunities and challenges. Eur Polym J. 2023;193: 112111.

[204] Tajaldeen A, Alrashidi M, Alsaadi MJ, Alghamdi SS, Alshammari H, Alsleem H, Jafer M, Aljondi R, Alqahtani S, Alotaibi A. Photoacoustic imaging in prostate cancer: a new paradigm for diagnosis and management. Photodiagnosis Photodyn Ther. 2024;47: 104225. 38821240 10.1016/j.pdpdt.2024.104225

[205] Grebinyk A, Chepurna O, Frohme M, Qu J, Patil R, Vretik L, Ohulchanskyy T. Molecular and nanoparticulate agents for photodynamic therapy guided by near infrared imaging. J Photochem Photobiol C Photochem Rev. 2024;58: 100652.

[206] Zhang S, Dong H, Bian J, Li D, Liu C. Targeting amyloid proteins for clinical diagnosis of neurodegenerative diseases. Fundam res. 2023;3:505–19. 38933553 10.1016/j.fmre.2022.10.009 PMC11197785

[207] Takeda Y. Modulating the photophysical properties of twisted donor–acceptor–donor π-conjugated molecules: effect of heteroatoms, molecular conformation, and molecular topology. Acc Chem Res. 2024. 10.1021/acs.accounts.4c00353. 39046948 10.1021/acs.accounts.4c00353 PMC11308373

